# Respiratory syncytial virus M2-1 protein associates non-specifically with viral messenger RNA and with specific cellular messenger RNA transcripts

**DOI:** 10.1371/journal.ppat.1009589

**Published:** 2021-05-18

**Authors:** Molly R. Braun, Sarah L. Noton, Emmeline L. Blanchard, Afzaal Shareef, Philip J. Santangelo, W. Evan Johnson, Rachel Fearns

**Affiliations:** 1 Department of Microbiology, Boston University School of Medicine; National Emerging Infectious Diseases Laboratories, Boston University, Boston, Massachusetts, United States of America; 2 Wallace H. Coulter Department of Biomedical Engineering, Georgia Institute of Technology and Emory University, Atlanta, Georgia, United States of America; 3 Division of Computational Biomedicine and Bioinformatics Program and Department of Biostatistics, Boston University, Boston, Massachusetts, United States of America; Washington University in Saint Louis, UNITED STATES

## Abstract

Respiratory syncytial virus (RSV) is a major cause of respiratory disease in infants and the elderly. RSV is a non-segmented negative strand RNA virus. The viral M2-1 protein plays a key role in viral transcription, serving as an elongation factor to enable synthesis of full-length mRNAs. M2-1 contains an unusual CCCH zinc-finger motif that is conserved in the related human metapneumovirus M2-1 protein and filovirus VP30 proteins. Previous biochemical studies have suggested that RSV M2-1 might bind to specific virus RNA sequences, such as the transcription gene end signals or poly A tails, but there was no clear consensus on what RSV sequences it binds. To determine if M2-1 binds to specific RSV RNA sequences during infection, we mapped points of M2-1:RNA interactions in RSV-infected cells at 8 and 18 hours post infection using crosslinking immunoprecipitation with RNA sequencing (CLIP-Seq). This analysis revealed that M2-1 interacts specifically with positive sense RSV RNA, but not negative sense genome RNA. It also showed that M2-1 makes contacts along the length of each viral mRNA, indicating that M2-1 functions as a component of the transcriptase complex, transiently associating with nascent mRNA being extruded from the polymerase. In addition, we found that M2-1 binds specific cellular mRNAs. In contrast to the situation with RSV mRNA, M2-1 binds discrete sites within cellular mRNAs, with a preference for A/U rich sequences. These results suggest that in addition to its previously described role in transcription elongation, M2-1 might have an additional role involving cellular RNA interactions.

## Author summary

RSV is a significant cause of respiratory disease in infants and the elderly population. As yet there are no vaccines or effective antiviral treatments to control the virus. An understanding of the viral machinery that generates viral RNA could help identify suitable targets for antiviral inhibitors. Our goal in this study was to elucidate how a key protein in RSV mRNA transcription, M2-1, functions by determining if it binds to specific RSV RNA sequences. Our findings show that while M2-1 binds RSV mRNAs, it makes contacts along the entire length of each mRNA, indicating that it is part of the RNA generating machinery. Surprisingly, we also found that M2-1 binds to specific cellular mRNAs. This finding suggests that M2-1 might have another role in infection, besides its role in RSV mRNA transcription.

## Introduction

Human respiratory syncytial virus (RSV), recently renamed as human orthopneumovirus [1,2] is the leading cause of pediatric acute lower respiratory tract infection. It has been estimated to cause approximately 3.2 million hospital admissions and 59,600 deaths per annum in children younger than 5 years [3]. Most children are infected before the age of two and reinfection can occur throughout life [4–6]. RSV also is a major pathogen among the elderly population and typically causes 11% of pneumonia-related hospitalizations in patients over 65 years of age and 14,000 deaths per year in the United States [7,8]. Currently, there is no licensed vaccine or effective antiviral therapy.

RSV is a member of the order *Mononegavirales*, a group of viruses with a single-strand negative-sense RNA genome. RSV transcription and genome replication occur in the cytoplasm of the cell [9]. These processes are performed by the viral RNA dependent RNA polymerase, which is a complex of at least two viral proteins, the large polymerase subunit, L, and the phosphoprotein, P [10,11]. Throughout the viral replication cycle, the genome exists as a nucleocapsid, in which the RNA is encapsidated along its length with oligomeric nucleoprotein (N) [12,13]. It is thought that the N-oligomer chain is transiently displaced by the polymerase to allow the template RNA to enter the polymerization active site, and then re-associates with the RNA as it emerges from the polymerase [14]. During transcription, the polymerase initiates RNA synthesis near the 3´ end of the genome [15,16]. Following recognition of the gene start signal at the beginning of the gene, the polymerase initiates mRNA synthesis, caps the nascent mRNA strand, and elongates the nucleotide chain until it reaches the end of a gene [17–19]. At the gene end, the polymerase polyadenylates the mRNA by reiterative stuttering on a U-tract in a gene end signal sequence, and releases the transcript [18–20]. The polymerase then scans to the next gene start sequence to transcribe the next gene [21]. In this way, the polymerase sequentially transcribes monocistronic viral mRNAs. During replication, the polymerase initiates at the 3´ end of the genome and disregards the gene start and gene end signals to synthesize the antigenome [22]. The antigenome contains a promoter at its 3´ end that signals genome synthesis [23–25]. The antigenome and genome RNAs are encapsidated as they are synthesized [26] and this is thought to increase polymerase processivity, enabling it to synthesize the full-length antigenome and genome RNAs.

A key factor in RSV transcription is a viral protein called M2-1. M2-1 is not required for the polymerase to initiate mRNA synthesis or terminate at a gene end signal [27]. It is not required for the polymerase to produce a short translatable mRNA, indicating that it is not required for mRNA capping [28], neither does it appear to be required for polyadenylation [29]. However, M2-1 is required for the polymerase to synthesize mRNAs longer than approximately 500 nt in length, and reach the subsequent gene, indicating that it acts as a transcription elongation factor [27,30]. M2-1 also results in accumulation of di- or multi-cistronic mRNAs, indicating that it causes the polymerase to read through gene end signals [27,29]. However, it should be noted that one of the initial studies that showed this was inadvertently performed with a mutant polymerase that produced abnormally high levels of readthrough mRNAs, whereas wt polymerase reads through gene junctions at a relatively low frequency, irrespective of M2-1 [27,31]. Together, these studies indicate that the major role of M2-1 during transcription is to facilitate intragenic elongation as the polymerase transcribes RNA from the gene start to gene end signals. Although required for RSV transcription, M2-1 was found to have no effect on RNA replication [27]. This is consistent with the finding that M2-1 immunoprecipitated from infected cells appears to be predominantly bound to RSV mRNAs [32].

M2-1 has been extensively characterized using biochemical and structural studies. The protein has three domains separated with flexible linkers [33,34]. Near the N-terminus is a cysteine-cysteine-cysteine-histidine (CCCH) Zn^2+^ finger motif necessary for RNA binding and elongation function [32–36]. The central region contains an oligomerization domain that allows the protein to form a symmetrical tetramer, followed by a core domain that also has RNA binding properties [33,34,37–39]. It is thought that M2-1 is recruited to the transcribing polymerase through tetramer-tetramer interactions with P [28,35,38–40]. Biophysical analysis of RSV M2-1 has shown that the protein can undergo structural transitions between a closed and open conformation, which could potentially enable functional modulation [41,42]. Intriguingly, the M2-1 protein of a closely related virus, human metapneumovirus (HMPV), has similar structural and dynamic features as that of its RSV counterpart, but in contrast to RSV M2-1, HMPV M2-1 is not required for viral transcription or for HMPV to replicate efficiently in cell culture [43,44]. Nonetheless, HMPV M2-1 does play an important role during infection as it is absolutely required for viral replication in an animal model [43]. These findings imply that RSV M2-1 might have more than one function during infection.

Several studies have been performed to determine if M2-1 has affinity for particular RNA sequences. Experiments to measure the binding properties of purified M2-1 protein and RNA oligonucleotides showed that M2-1 has a greater affinity for A-rich oligonucleotides and oligonucleotides containing the complement of the RSV gene end signal sequence (i.e. positive sense gene end sequences) [34,37]. Given its affinity for A-rich sequences, it was suggested that M2-1 may assemble with gene end sequences and poly(A) tail [34,37], perhaps to exert a post-transcriptional role [38,45,46]. However, this model does not explain how M2-1 functions to facilitate elongation *within* genes, i.e. before the polymerase reaches the gene end signal [21,28,30]. Furthermore, another study showed that M2-1 binds specifically to the complement of the leader promoter sequence that precedes mRNAs, and non-specifically to longer RNAs [35], and another showed that M2-1 protomers bind cooperatively to RNA, enabling M2-1 tetramers to bind to RNA with high affinity but low specificity [47]. Thus, the data obtained using *in vitro* RNA binding assays have yielded somewhat conflicting data.

Given the apparent discrepancies between the different biochemical studies, the goal of this study was to identify RNA sequences contacted by M2-1 in RSV infected cells. This question was addressed by performing a CLIP-seq analysis of M2-1 bound RNA. Our results show that M2-1 associates with viral mRNA across the entire length of each gene, consistent with M2-1 contacting each nucleotide of nascent mRNA being extruded from the polymerase. In addition, we discovered that M2-1 binds to specific sites within a subset of cellular mRNAs, with a bias towards adenine and uracil containing sequences. These findings help reconcile the previously published data and suggest that M2-1 has two RNA-binding functions in RSV infection, functioning as a viral transcription elongation factor, and binding to specific cellular mRNAs.

## Results

### Optimization of the CLIP-Seq protocol for analyzing RSV M2-1: RNA complexes

**Fig 1:**
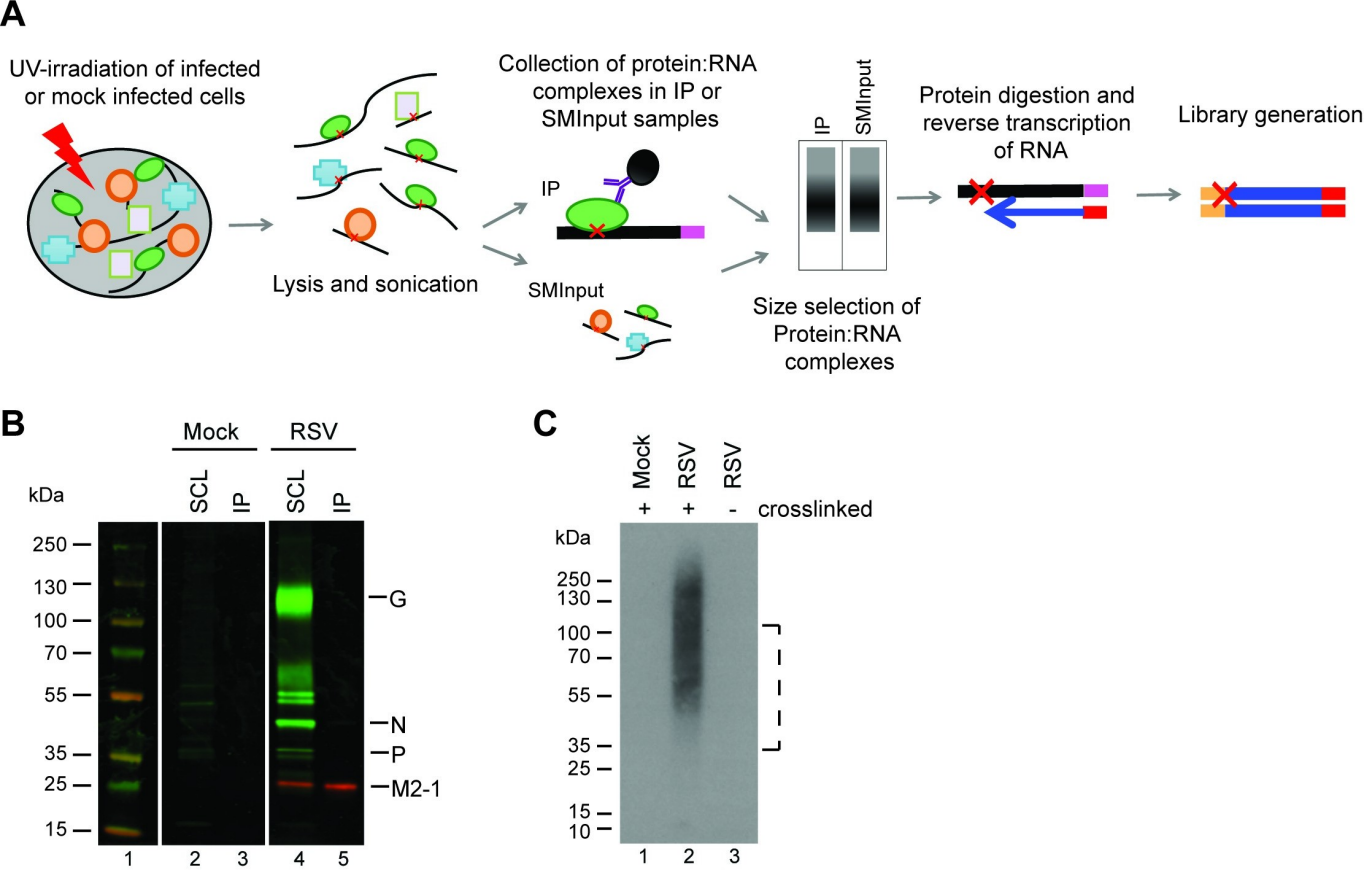
Experimental design to identify RNA bound by M2-1. (A) Overview of seCLIP-seq procedure. RSV infected cells were subjected to UV-irradiation to crosslink RNA and protein complexes. Cells were lysed and RNA sheared by sonication. A subsample of lysate was removed for the SMInput control while the remaining was subjected to immunoprecipitation using an M2-1 specific antibody. Protein: RNA complexes were separated by size by SDS-PAGE and transferred to a nitrocellulose membrane. RNA was released from the membrane by proteinase digestion, reversed transcribed to the site of the protein:RNA crosslink (indicated with a red X), and used to generated a library, which was analyzed by sequencing. (B) Western blot analysis of RSV proteins immunoprecipitated from infected cells by the anti-M2-1 22k4 antibody following UV-cross linking. Soluble cell lysates (SCL) and immunoprecipitated complexes (IP) were analyzed by Western blotting using a polyclonal anti-RSV antibody (green) and an M2-1 specific antibody (red). (C) Visualization of RNA immunoprecipitated in complex with M2-1. Cells were mock infected or infected with RSV A2 and subjected to UV light cross-linking (40 mJ/cm^2^) or not, as indicated. Cell lysates were treated as described above, except that immunoprecipitated RNA was radio-labeled prior to SDS-PAGE, transferred to nitrocellulose membrane, and analyzed by autoradiography. The dotted line indicates the region that was excised in the subsequent seCLIP-seq analysis (which entailed unlabeled RNA).

For the CLIP-seq analysis we employed single-end enhanced CLIP-seq (seCLIP-seq) [48], a technique that can resolve protein:RNA binding at individual nucleotide resolution. An overview of the procedure we used is presented in Fig 1A. To immunoprecipitate M2-1 for the CLIP-seq and subsequent validation experiments we used M2-1 specific monoclonal antibodies, 22k4 and 37M2. Both antibodies were characterized in detail previously and bind to epitopes in the C-terminal domain of M2-1 [49]. Because M2-1 is thought to be a component of a transcriptionally active nucleocapsid it was necessary to identify conditions that would allow isolation of M2-1: RNA complexes, without co-precipitating RSV nucleocapsids or nucleocapsid fragments. This is because nucleocapsids would also contain N-associated genome and antigenome RNA, which would obscure the analysis. Therefore, we tested different UV exposures and RNAse digestion conditions. We found that UV exposure of 40 mJ/cm^2^ (rather than 400 mJ/cm^2^) minimized co-precipitation of RSV N and P proteins (S1 Fig). RNase digestion is typically performed following sonication to further digest RNA and help improve binding site resolution. However, we found that while shearing by sonication-only allowed us to purify M2-1 away from any other RSV proteins, inclusion of a nuclease digestion step resulted in co-precipitation of RSV N protein (S1 Fig). Omission of the RNase digestion step allowed us to minimize co-precipitation of RSV N or P proteins (Fig 1B). One drawback to omitting the nuclease digestion procedure is that this could bias the analysis towards more accessible M2-1: RNA complexes and might provide less information regarding M2-1: RNA interactions in larger ribonucleoprotein complexes, such as RSV inclusion bodies. Therefore, in one experimental replicate (replicate 1), we omitted RNase inhibitor from the immunoprecipitation step, allowing for limited RNAse digestion by cellular nucleases. This did not lead to co-precipitation of RSV N or P protein, but did lead to slightly sharper peaks in the seCLIP-seq analysis and some differences in the results regarding interactions with RSV RNAs, as described in more detail below. Finally, we confirmed that the 22k4 antibody that was used for the CLIP-seq experiment did not cross-react with cellular RNA binding proteins (or RNAs). Radiolabeling of RNA co-precipitated by M2-1 showed that RNA was present specifically in RSV-infected, UV-crosslinked samples (Fig 1C). This indicated that the 22k4 antibody did not bind appreciably to cellular RNA binding proteins or RNA, and that the conditions used allowed for specific isolation of RNA in direct contact with M2-1 at the time of cross-linking.

### M2-1 binds to RSV mRNAs

RSV-infected A549 cells were exposed to UV light at 8 and 18 hpi. These time points were selected as representatives of the early and late times in infection. M2-1:RNA complexes were isolated and cDNA libraries generated, as indicated in Fig 1A, using the experimental parameters described above. A size-matched input (SMInput) control was prepared in parallel. This consisted of an aliquot of the same lysate, removed immediately prior to the M2-1 immunoprecipitation step, and then subjected to the same size selection and library preparation steps (Fig 1A). Comparison of sequences obtained in the immunoprecipitated (IP) sample versus the SMInput control allowed identification of RNA sequences that were enriched by M2-1 immunoprecipitation. Two biological replicates were performed at 8 hpi and 18 hpi (replicates 1 and 2), and additional replicate at 18 hpi (replicate 3); a mock infected control was performed for each time point.

**Fig 2:**
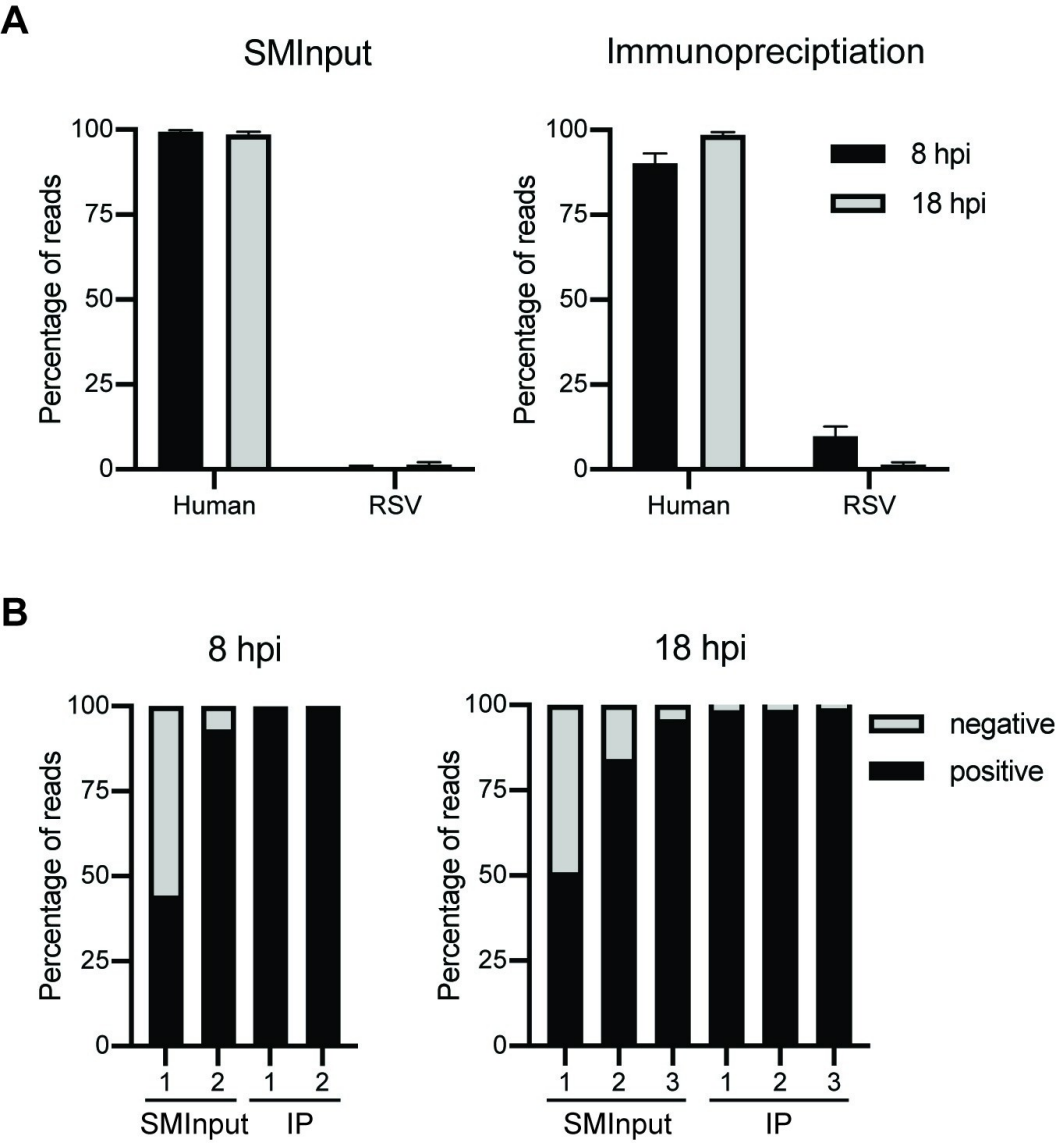
M2-1 binds to cellular and positive sense RSV RNA. (A) Genomic identity of RNA bound by M2-1 at 8 (black) and 18 (grey) hpi. Libraries were aligned to human (hg19) and RSV (NCBI Accession # M74568.1) genomes. Reads that did not map to either of these genomes (for example, poly(A) tails, contaminants from the air and nitrocellulose etc.) were discarded in this analysis, as were ribosomal RNAs and repetitive sequence elements. Analysis was done for SMInput and M2-1 IP samples (left and right panels, respectively). The mean average value of the two replicates at 8 hpi and three replicates at 18 hpi is presented. (B) Percentage of negative (grey) versus positive (black) sense RSV RNA from 8 hpi and 18 hpi (left and right panels, respectively). Each individual replicate is represented.

IP and SMInput libraries were aligned to the human genome (hg19) and RSV A2 genome (NCBI Accession # M74568.1; S1 Table). Surprisingly, M2-1 was found to associate with both human and RSV RNA, with an increase in the amount of human versus RSV RNA at 18 versus 8 hpi in each of the replicates (Fig 2A). It should be noted that the relative proportions of human and RSV RNA identified in the seCLIP-seq analysis might not accurately represent the proportions of RNA bound by M2-1 in infected cells because RNA sequestered in insoluble complexes would have been removed prior to the immunoprecipitation step. Nonetheless, clearly RSV M2-1 binds to human as well as viral RNAs. It has previously been reported that M2-1 binds preferentially to RSV mRNAs, as opposed to RSV genome (or antigenome) RNAs [32]. To determine if the CLIP-seq data represented what has previously been described, the RSV reads were further evaluated to determine if they were of positive or negative sense polarity. The RSV RNAs within the replicate SMInput samples contained 4.2–55.7% negative sense RNA and 44.3–95.7% positive sense RNA (Fig 2B). The replicate 1 SMInput samples contained a greater proportion of negative sense RNA, likely because of partial digest of the positive sense mRNA in these samples. In contrast to the SMInput samples, the RSV RNAs within all the IP samples were 98.5–99.6% positive sense (Fig 2B). Given that only a very small fraction of positive sense RNA is antigenome, and that genome RNA was not precipitated by M2-1, this result is consistent with previous findings that showed that M2-1 binds to RSV mRNAs rather than RNA replication products.

### M2-1 makes contacts along the entire length of RSV mRNAs

**Fig 3:**
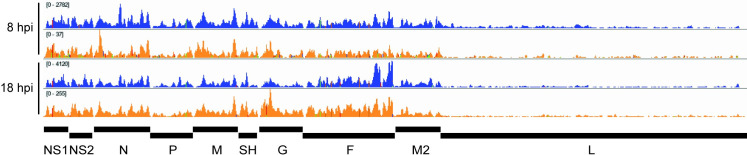
Read coverage of the RSV genome in SMInput and IP samples. Read coverage tracks of positive sense RSV reads for IP (blue traces) and SMInput (orange traces) in the 8 and 18 hpi replicate 2 samples. Images were generated using IGV. Note that the *y*-axes are scaled differently for each track to allow direct comparison. Read tracks for the other replicate samples are presented in S2 Fig.

**Fig 4:**
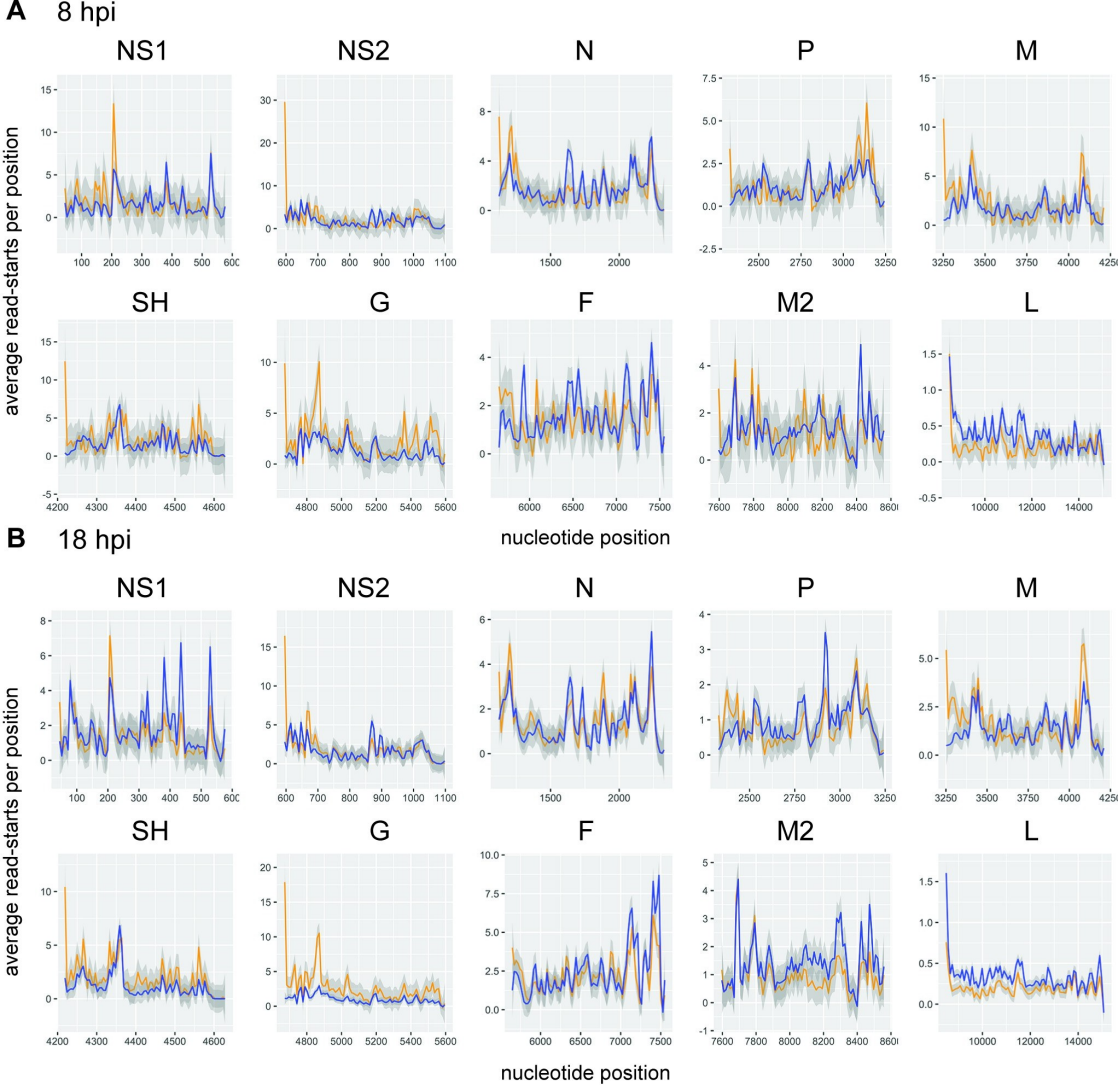
There are M2-1 cross-link sites along the RSV transcriptome. Percent occurrence of read end sites at each nucleotide position within each RSV gene (positive sense) were plotted with the IP read end sites shown in blue and the SMInput read end sites in orange. Graphs represent the average values per position for the 8 (n = 2) and 18 (n = 3) hpi (panels A and B, respectively). The grey shading represents the standard error between the different replicates.

**Fig 5:**
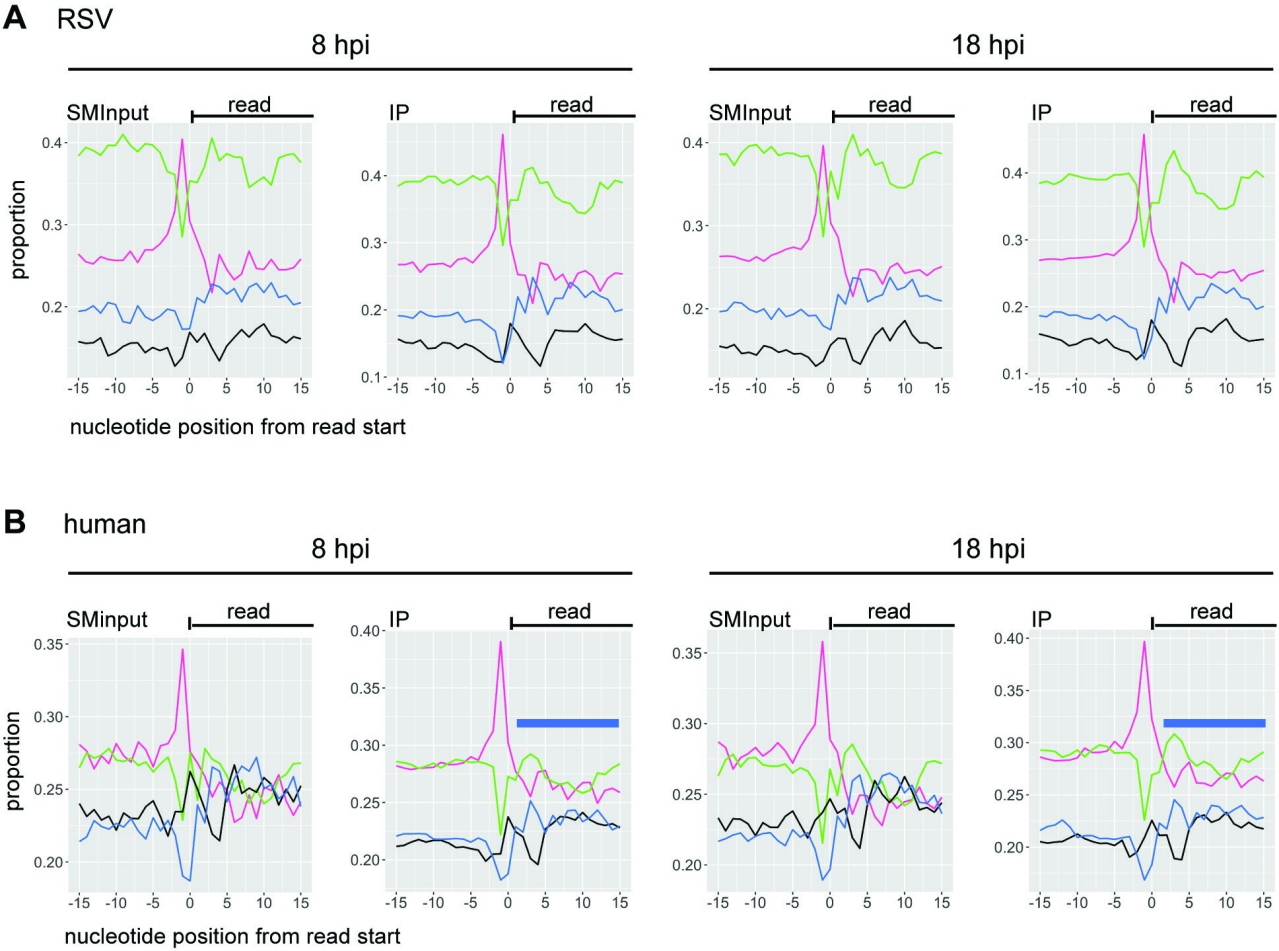
Nucleobase composition adjacent to cross-link sites. (A and B) Frequency of each nucleotide flanking the point of crosslinking for RSV (A) and human (B) aligned reads. The *x*-axis represents the nucleotides flanking the read end (position 0) and the *y*-axis represents the frequency of each nucleotide identity at the given positions. The different lines represent A (green), U (magenta), G (black) and C (blue). Graphs represent the average values per position for the combined 8 (n = 2) and 18 (n = 3) hpi samples. The blue bars in panel B indicate the region of AU-enriched sequence in the M2-1 IP samples.

Having found that M2-1 associates selectively with RSV mRNAs, rather than genome RNA, we examined if it binds to specific sequences. First, we compared the read alignments in the M2-1 IP samples to the SMInput. This analysis showed that for each of the replicate samples, the depth of coverage pattern for the M2-1 enriched samples was similar to the SMInput control, indicating that M2-1 could bind the length of RSV mRNAs (Figs 3 and S2). Further, during the cDNA preparation step performed to generate the sequencing library, reverse transcriptase terminates at the position preceding a protein: RNA cross-link site. Thus, the start of each RNA-seq read may represent a site proximal to protein: RNA binding. It would be expected that cross-link sites would occur along the length of each RSV mRNA in both the SMInput and IP samples due to mRNA interaction with cellular proteins (e.g. ribosomal proteins, decay factors etc.) and viral proteins (e.g. polymerase), and indeed this is what was found (Fig 4). However, if M2-1 were to bind to a particular sequence within RSV mRNAs, it would be expected that cross-link sites would be enriched in that sequence region in the IP sample, as compared to the SMInput control. To investigate if this was the case, t-tests were performed at each position along each gene to determine if there were significantly more cross-link sites in the IP samples as compared to the SMInput at each given position. The t-tests were performed separately for the 8 hpi and 18 hpi samples. Although a few scattered points within each gene were found to be significant, when p-values were adjusted for multiple t-tests controlling for false discovery rate, no points were significant (*p* ≥ 0.05). This indicated that there was no enrichment of any particular M2-1 binding site in RSV mRNAs at either time point. Finally, we also analyzed the nucleotide content surrounding the read start sites from our library preparations. Although UV-C crosslinking can induce crosslinks between any nucleotide and the protein to which it is bound, cross-linking occurs with a greater frequency at uridine residues [50,51]. Consistent with this, we found that position -1 relative to read starts was enriched in uridines, indicating that the majority of read-ends corresponded to nucleotides adjacent to cross-linking sites (rather than sites of RNA cleavage, or premature reverse transcriptase termination) (Fig 5A). If M2-1 were to bind a specific RSV RNA sequence, we would expect to see that the relative levels of those nucleobases enriched in the nucleotides proximal to the crosslink sites in the immunoprecipitation samples, but not the SMInput samples. However, comparison of the SMInput and IP samples showed similar patterns of nucleobase distribution at the nucleotides flanking the point of RNA crosslinking, indicating that isolation of M2-1 bound RNA did not result in specific enrichment of any particular RSV RNA sequence (Fig 5A).

**Fig 6:**
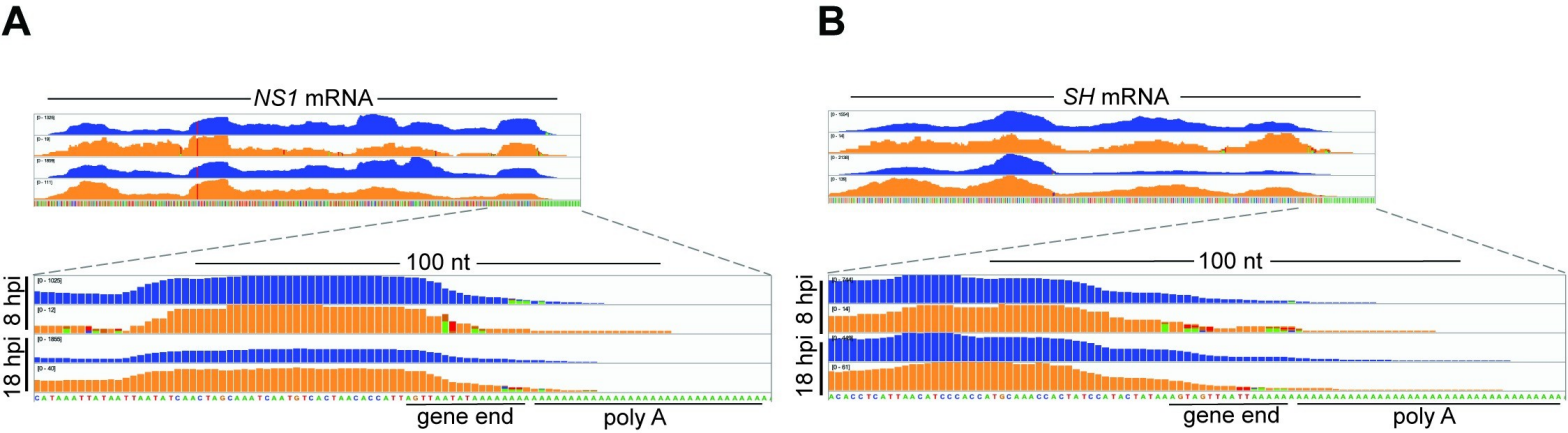
M2-1 does not bind with a higher density at RSV gene end sequences than at intragenic regions. Read coverage tracks of positive sense RSV reads aligned to RSV *NS1* (A) and *SH* (B) mRNA sequences containing a 41 nt polyadenylate sequence added to the 3´ end. The tracks show IP (blue) and SMInput (orange) traces for the 8 and 18 hpi replicate 2 samples. The positions of the positive-sense gene end and polyadenylate sequences are indicated. Note that the sequence is written as positive sense DNA.

Previous studies using short RNA oligonucleotides indicated that that M2-1 has affinity for the positive sense gene end and polymeric A sequences that would be present at the 3´ end of each mRNA [34,37]. The affinity differs depending on whether M2-1 is monomeric or tetrameric, but in both cases, the affinity for the positive sense SH gene end signal and an A rich sequence was similar. In the case of monomeric M2-1, the dissociation constants for the SH gene end and a poly-A rich sequence were found to be 13 and 22 μM, respectively [37], whereas in the case of tetrameric M2-1, they were 46.5 nM and 19.1 nM, respectively [34]. Based on this, it has been suggested that M2-1 might bind to RSV gene end sequences and/or the poly(A) tail. It is not possible to mine the CLIP-seq data to determine if M2-1 was binding preferentially to RSV poly A tail sequences beyond the gene end sequence for a number of technical and scientific reasons (see Discussion). However, binding to the positive sense gene end sequences (which include a poly A tract) could be investigated. The mRNA poly A tails were not represented in the RSV reference sequence used in the alignments used in Figs 3 and 4, meaning that if M2-1 bound to the gene end sequences in such a way that the cross-link site was very close to or immediately adjacent to the poly A tail, we would not have detected those reads or cross-link sites. Therefore, to determine if M2-1 has a propensity for binding to RSV positive-sense gene end sequences, we aligned the reads to RSV *NS1* and *SH* mRNA sequences that had a 41 nt poly A tail added following the gene end signal. This analysis showed that the read coverage profiles in the gene end region were similar between the IP and SMInput samples, demonstrating that M2-1 did not preferentially bind to the positive sense gene end sequences that lie at the end of each mRNA (Fig 6).

### M2-1 shows evidence for associating with mRNA in conjunction with the transcriptase complex

**Fig 7:**
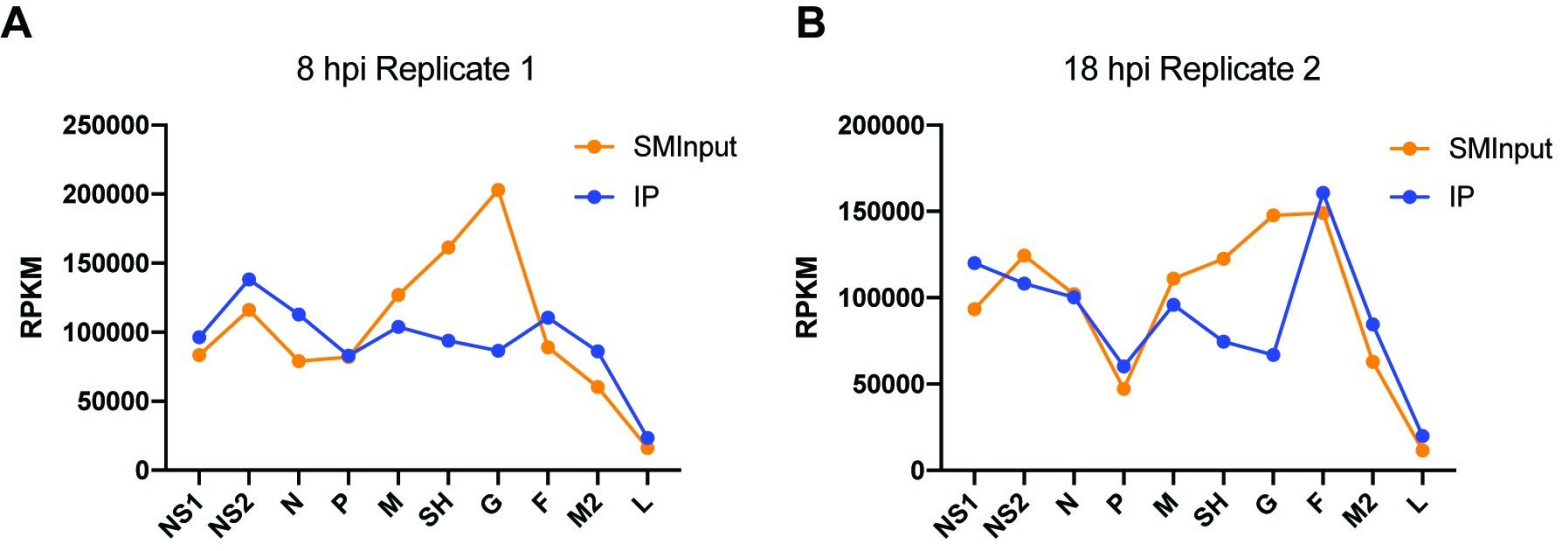
M2-1: RNA interactions show evidence of a transcription gradient. RPKM values were calculated for RSV positive sense sequences represented in the SMInput and IP datasets and plotted against each of the RSV genes. The figure shows graphs for the 8 hpi replicate 1 (A) and 18 hpi replicate 2 samples (B); graphs for the other samples are presented in S3 Fig.

The data presented above show that M2-1 can bind the entire RSV transcriptome. These data suggest two possible models for M2-1 interactions with RSV transcripts. The first is that M2-1 associates with RNA being extruded from the polymerase, such that M2-1 transiently contacts each nucleotide of RSV mRNAs. The other possibility is that M2-1 associates with RSV mRNAs post-transcriptionally, in a stochastic manner, such that no particular binding site is detected. Transcripts that were in the process of being synthesized at the time of UV-crosslinking would be expected to follow the RSV transcription gradient, with genes at the 3´ end of the genome being represented at higher levels than those at the 5´ end. In contrast mRNAs in the post-transcriptional state at the time of UV-crosslinking would be present at a level that reflects both transcription frequency and transcript stability. These steady state mRNAs would be reflected in the SMInput datasets. To distinguish between co-transcriptional and post-transcriptional binding of M2-1 to RSV mRNAs, we examined the reads per kilobase of transcript, per million mapped reads (RPKM) values for each of the RSV genes in the IP and SMI RNA datasets. Comparison of the RPKM values for the IP and SMInput RNA libraries in the 8 hpi replicate 1 sample showed a clear divergence. Whereas the SMInput RNA showed variable levels of each mRNA species, with relatively high levels of M, SH and G compared to the other genes, the IP RNA from the same experiment showed a pattern more closely aligned with a transcription gradient (Fig 7A). These data suggest that the majority of M2-1: RNA contacts that were detected in this replicate and time point were occurring in the process of transcription. The 18 hpi replicate 2 sample showed a slightly different pattern. In this case, the SMInput RNA libraries showed an under-representation of *P* reads compared to *M*, *SH*, *G* and *F*, suggesting that *P* mRNA is relatively unstable in the cellular milieu. The IP RNA showed a transcription gradient, similar to that of the 8 hpi replicate 1 sample, but with a prominent under-representation of *P* and an over-representation of *F* mRNAs (Fig 7B). This indicates that in this sample, M2-1 was contacting mRNA in the process of being transcribed, and might also have been associated with RNA following transcription, where mRNA steady state levels would also come into play. We reason that the difference between the 8 hpi replicate 1 and 18 hpi replicate 2 profiles is due to the fact that at 8 hpi the proportion of RSV mRNA being actively transcribed versus in a post-transcriptional phase would be relatively high as compared to 18 hpi, when much of the mRNA in the cell would have been generated earlier in infection. In addition, the limited nuclease digestion that occurred in the preparation of the replicate 1, but not replicate 2, sample might have helped to release nucleocapsids and render M2-1 protein involved in transcription more accessible for immunoprecipitation. Consistent with this, the RPKM profile in the 18 hpi replicate 3 sample is almost identical to that shown in Fig 7B, and the other two samples (8 hpi replicate 2 and 18 hpi replicate 1) show an intermediate pattern (S3 Fig).

### M2-1 binds to specific cellular mRNAs

**Fig 8:**
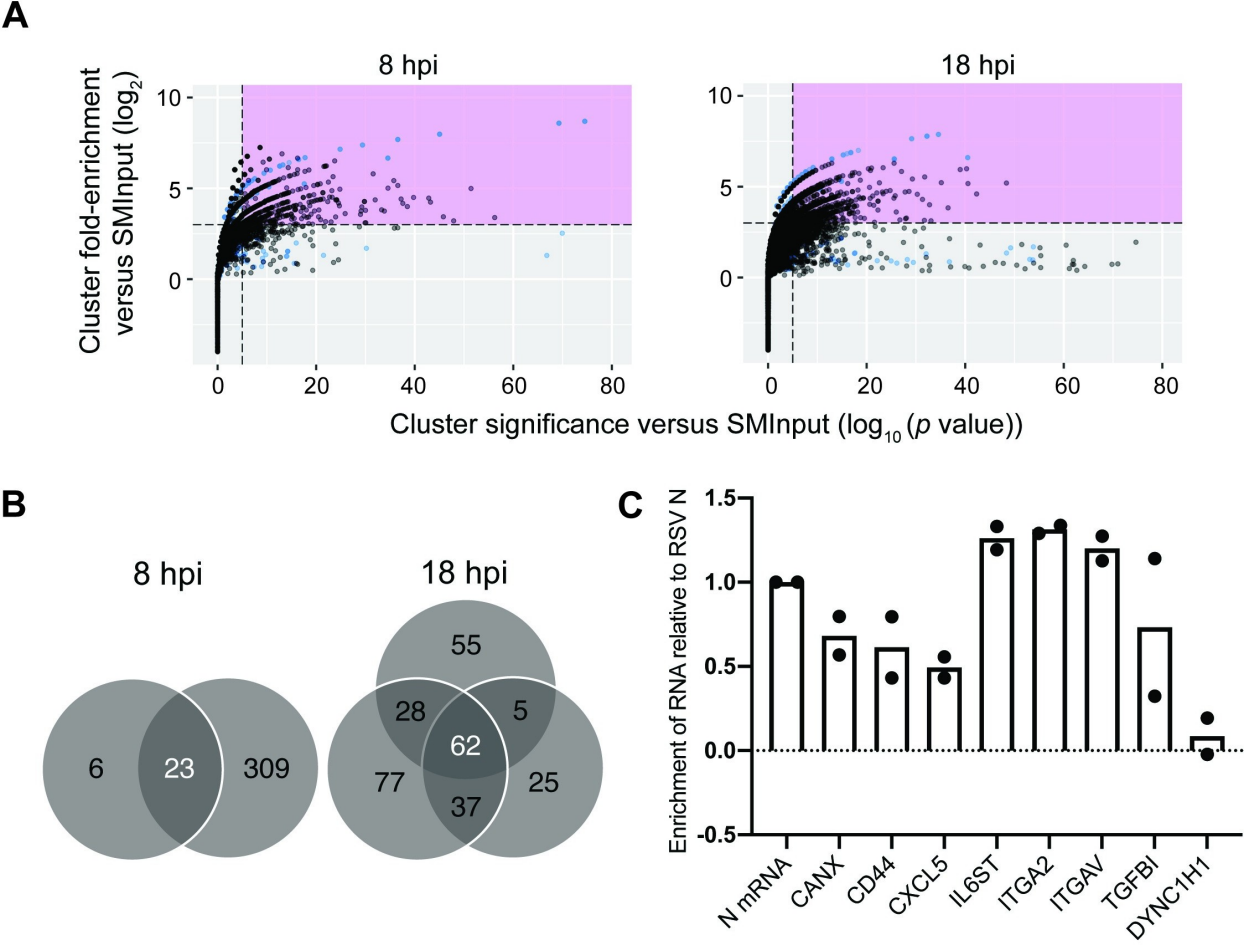
M2-1 binds specifically to host cell RNA. (A) CLIPper-identified peaks were graphed for fold enrichment vs significance using ggplot2. Peaks within the region identified in pink were considered significant based on eCLIP standards. All peaks were plotted from each sample for 8 and 18 hpi for RSV infected (black) and mock infected (blue). (B) Gene identities of significant peaks were determined and common genes between samples were identified. (C) RNA immunoprecipitation followed by RT-qPCR of target genes identified by CLIPper. Levels of co-immunoprecipitated RNA were normalized to levels of co-immunoprecipitated *N* mRNA. Individual data points for two biological replicate experiments are shown.

The data presented in Fig 2A show that while M2-1 binds to RSV RNAs, cellular RNAs were also present in the M2-1 IP samples, with an increase in cellular relative to viral RNAs later in infection. We sought to understand if M2-1 binds specific cellular RNAs. To perform this analysis, CLIP-seq reads that mapped to the human genome were analyzed by the peak-finding program CLIPper, which compares the reads found in the IP versus SMInput cDNA libraries and determines the fold change of sequence reads within the region of interest, and the *p*-value of each peak, between the IP and SMInput samples [52]. Each peak from the two 8 hpi and three 18 hpi samples (black), as well as the peaks identified from the mock infection (blue) was given a score based on these two attributes and plotted (Fig 8A). Peaks that were enriched at least 8-fold with *p* ≤ 10^−5^ compared to SMInput were considered significant [53]. Each significant peak was mapped to a human gene. The peaks identified in the mock infected samples represent RNA sequences associated with cellular proteins that bound non-specifically to the antibody or beads during immune precipitation. Therefore, we next identified genes that were represented in multiple samples from RSV infected cells, but not in the samples from mock infected cells. We found 23 genes in common between the two 8 hpi samples, 62 in all three of the 18 hpi samples, and 12 genes were identified in all five (8 + 18 hpi) samples (Fig 8B and Table 1; *SCD* was identified in the samples from mock infected cells, but a different gene region was identified as a peak in all five RSV infected cell samples and so *SCD* is also included in this list of genes.) There was even more overlap between the different samples if we relaxed the criteria and considered genes that were not represented in all replicates at each time point. For example, comparison of the 8 hpi replicate 2 samples with the three 18 hpi samples showed 54 genes in common. Conversely, of the 11 genes represented as being in common in both 8 hpi replicates, but not all three 18 hpi replicates in Table 1, seven were found in two of the 18 hpi replicates, and only one gene (*RPLP0*) was not found in any of the three 18 hpi replicates. Thus, there was considerable overlap in the genes identified at the different times post infection. Some of the identified genes were highly represented in the SMInput or have been categorized as abundant or housekeeping genes [54] and did not show evidence for the same peak in multiple samples. It is possible that these mRNAs were bound non-specifically by M2-1 because of their relatively high abundance in infected cells. However, in other cases, the same peaks were identified in multiple samples (indicated with asterisks in Table 1) and were identified in genes that are not considered abundant or housekeeping genes. These findings indicate that M2-1 specifically binds to a subset of cellular mRNAs.

**Table 1:** Genes identified by CLIPper analysis. The table shows genes that were identified as significant peaks by CLIPper in the two 8 hpi samples, the three 18 hpi samples, or all five samples. Genes with overlapping peak regions in different replicates within either the 8 or 18 hpi grouping are indicated with asterisks.

8 hpi (1+2)		18 hpi (1+2+3)					Shared
LAMC2*	RPL37A*	RPS8	SLC38A2*	TIMP2	TFRC	SEMA3C	SCD*
SCD*	DCBLD2	CAPN2	TMBIM6	DSG2	CXCL1	TFPI2	LDHA
LDHA*	TM4SF1*	VIM*	ATP2A2*	LMAN1	CXCL5*	MET*	CD44
CD44*	CXCL5*	ITGB1*	TMED2*	RPS19*	SLC7A11	SLC39A14*	ATP2A2
RPS3	RPS14	VCL	RPS29	PDIA6	OSMR*	TMEM66*	ZFP36L1*
PTGES3	CANX*	TM9SF3	CNIH1*	RTN4*	ITGA2	ADAM9*	RPS19*
ATP2A2	SEMA3C	SCD*	HIF1A*	ITGAV*	IL6ST	ASPH	DCBLD2
RPLP0	SERPINE1*	EIF4G2	ZFP36L1*	TFPI	MAP1B	RPL30	TM4SF1*
ZFP36L1*	AKR1B10*	LDHA*	B2M*	PRNP	NT5E	ANXA1	CXCL5*
HSP90AA1	SLC39A14*	CD44	PTPLAD1*	RHOA	TMED7	GOLM1	CANX*
SRSF1*	RPS19*	FTH1*	IQGAP1	DCBLD2*	TGFBI*	MAGT1	SEMA3C
ACTG1*		CNTN1	CLTC*	PLOD2*	CANX*		SLC39A14*
		SLC38A1	CCDC47*	TM4SF1*			

To validate a portion of these hits, cells were infected with RSV and at 18 hpi they were subjected to cross-linking and lysis using similar conditions as in the CLIP-seq experiment, except that we used a different preparation of RSV A2 and a different M2-1 specific monoclonal antibody, 37M2 [49], for immunoprecipitation. Following RNA: M2-1 immunoprecipitation, a selection of genes identified as being significant in the CLIPper analysis (including three of the mRNAs common to all five replicates) were quantified by RT-qPCR analysis and compared to RSV *N* mRNA, which would be expected to be associated with M2-1 protein. Dynein cytoplasmic 1 heavy chain 1 (*DYNC1H1*), which was highly represented in both the SMInput and IP samples, but was not identified as a significant gene using the CLIPper analysis was analyzed in parallel as a control. This analysis showed enrichment of the CLIPper identified genes compared to RSV *N* mRNA, with little or no enrichment of *DYNC1H1* (Fig 8C). These findings validate that M2-1 associates with specific cellular mRNAs.

### M2-1 is associated with specific regions within cellular mRNAs

**Fig 9:**
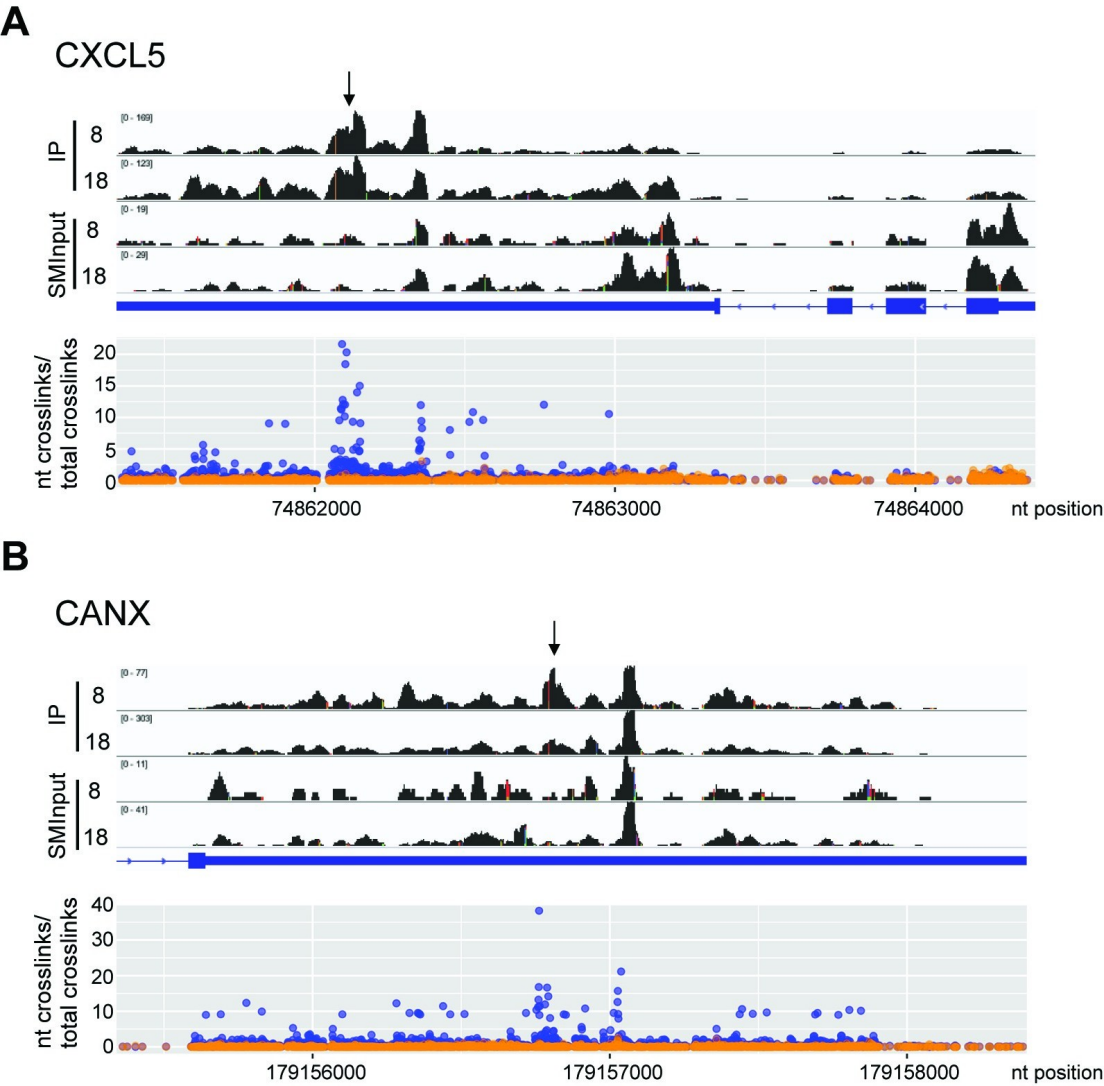
M2-1 binds specific regions of CXCL5 and CANX mRNAs. (A) The upper panel shows read coverage tracks of the *CXCL5* gene in M2-1 IP samples. A representative 8 and 18 hpi replicate is presented. Note that the *y*-axes are scaled differently for each track to allow direct comparison. The gene organization is shown below, with exons shown as thick blue lines, the untranslated regions as a medium blue line and introns as thin blue lines. Arrowheads on the blue lines represent the gene orientation. The lower panel shows the number of nucleotide crosslinks per genomic position within *CXCL5* for SMInput samples (orange) and IP samples (blue). The mean averages of all 5 replicates are represented. (B) Same as A, but for the relevant portion of the *CANX* gene (the remainder of the gene was omitted due to its large size). Black arrows represent the CLIPper identified peak that was common to all five samples.

Having found that M2-1 interacts with specific cellular mRNAs, we examined if it binds to specific sequences within those RNAs. As noted above, the CLIPper algorithm searches the datasets to identify sequence peaks that are enriched in the IP samples, compared to the SMInput controls. These peaks represent potential M2-1 binding sites. Therefore, we examined how many of the sequence peaks overlapped between multiple samples. We found that 15 of the 23 genes that were represented in both the 8 hpi samples, 29 of the 62 genes in all three of the 18 hpi samples, and 7 of the 12 genes that were identified in all five samples contained overlapping peaks (genes with overlapping peaks are marked with asterisks in Table 1). This indicates that M2-1 binds to specific regions within these mRNAs. We analyzed the seven genes with overlapping peaks in all five samples in more detail to identify the site of M2-1 binding in the mRNA. Comparison of the read coverage densities and read-ends in the IP and SMInput samples in these seven genes showed an enrichment of reads within specific regions of the 3´UTRs of six of the seven of these mRNAs. Because the CLIPper algorithm only takes sequence reads, but not read-ends (i.e. cross-link sites), into consideration, we also mapped the cross-link sites onto the two genes with the most significant CLIPper scores, *CANX* and *CXCL5*. This showed that there was clustering of cross-linking sites which correlated with the peaks identified by the CLIPper algorithm (Fig 9). These findings indicate that M2-1 associates with specific sites or regions within cellular mRNAs.

We examined if M2-1 binds specific RNA sequences within cellular mRNAs by examining the nucleobase composition adjacent to the read-ends (Fig 5B). As was seen in the case of RSV RNAs, the -1 position was predominantly a uridine residue, indicating that read-ends typically corresponded with cross-link sites, rather than non-specific reverse transcriptase termination. Analysis of the SMInput sample revealed that the sequence downstream of the cross-link site was an approximately even mix of A, C, G, and U residues and the sequence upstream was enriched in A and U residues. Given that the SMInput samples represent RNA bound to protein, but not M2-1 specifically, the enrichment in A and U residues upstream of the cross-link site presumably represents the binding preference of the majority of cellular RNA binding proteins. In the M2-1 IP samples a different pattern was seen. In the region upstream of the cross-link site, there was a subtle increase in the enrichment of A/U residues, compared to the SMInput control, but more strikingly there was an enrichment of A/U residues downstream of the cross-link site (the region indicated with a blue bar in Fig 5B). This finding suggests that M2-1 associates with AU rich sequences, consistent with what has been shown in biochemical assays [34,37].

### Cytoplasmic versus inclusion body associated M2-1 can be differentially detected with different antibodies

**Fig 10:**
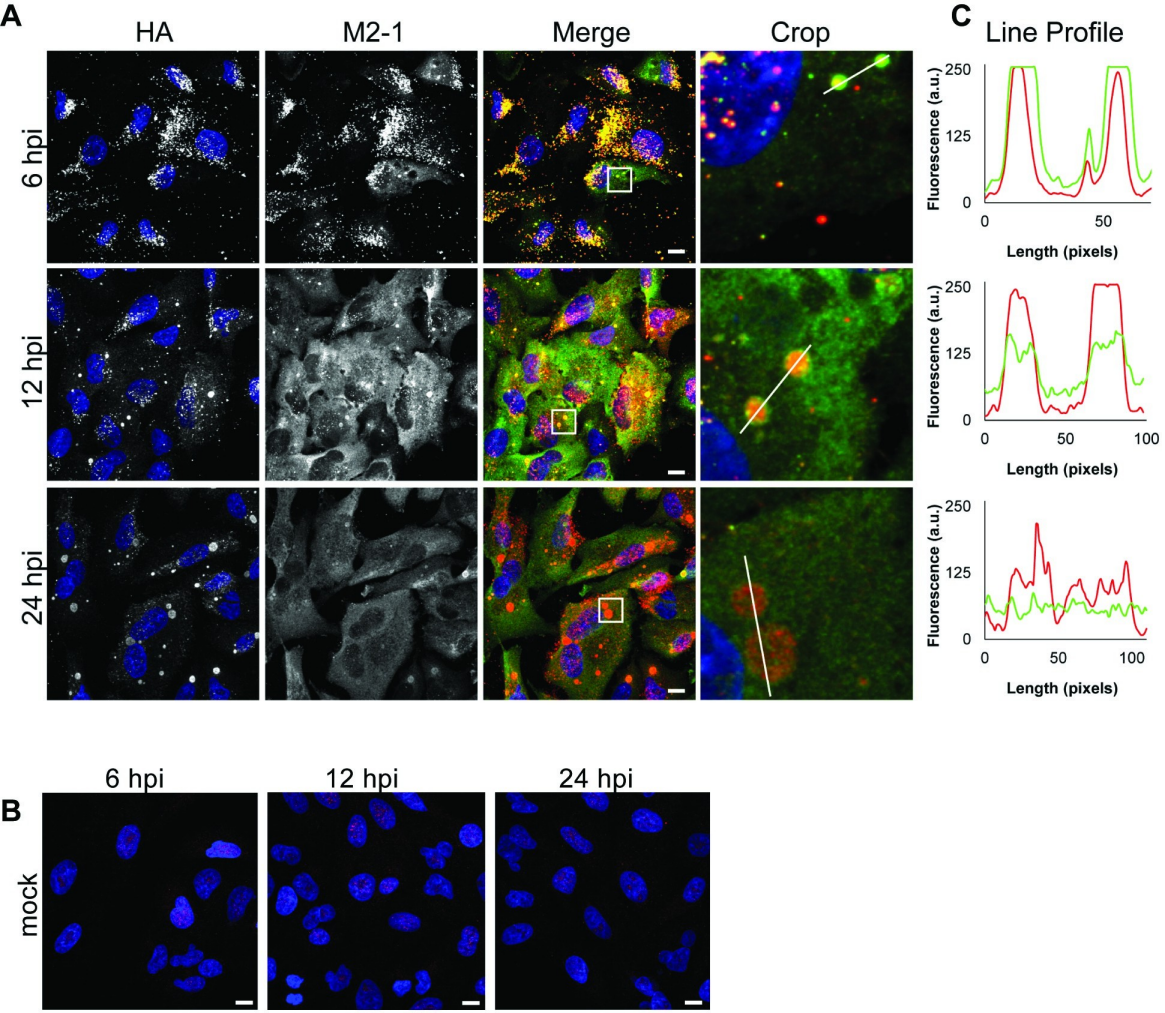
M2-1 localizes to viral inclusions and the cytoplasm at various times post infection. A549 cells were infected with rRSV_HA-M2-1_ at an moi of 3 pfu/cell (A) or mock infected (B) and fixed at 6, 12, and 24 hpi. A commercial antibody specific to M2-1, αM2-1 (green) and αHA (red) were used to visualize M2-1 at different times post infection; scale bars = 10 μm. (C) Line profile of M2-1 bound by the αM2-1 (green) or αHA antibody (red) drawn across the white line in the corresponding cropped images.

Finally, we examined the distribution of M2-1 in infected cells at different times post infection with different antibodies. It has previously been shown that in the early stages of infection, RSV nucleocapsid proteins (N, P, and L) exist in small puncta, which increase in size over the course of infection to form inclusion bodies. These puncta and inclusion bodies are the sites of transcription and genome replication [46,55,56]. M2-1 has been shown to localize in these puncta and inclusion bodies, within compartments within late stage inclusion bodies, referred to as IBAGS, which contain newly synthesized RSV RNAs, and more diffusely in the cytoplasm [37,56]. Analysis of A549 cells infected with a recombinant version of RSV containing an N-terminal HA-epitope tagged version of M2-1 (rRSV_HA-M2-1_) showed that both an anti-HA antibody and a commercially available M2-1 antibody detected M2-1 specifically (Fig 10A and 10B), but surprisingly, they revealed different distributions of M2-1, even though all the M2-1 present in the cells contained an HA tag. In cells fixed at an early time in infection (6 hpi), the two antibodies had a largely overlapping staining pattern, co-localizing in small puncta throughout the cytoplasm. At 12 hpi, the anti-HA antibody detected M2-1 predominantly in cytoplasmic inclusions. In contrast, the anti-M2-1 antibody detected M2-1 distributed diffusely in the cytoplasm, in addition to cytoplasmic inclusions. By 24 hpi, the distinction was even more pronounced, with the anti-HA antibody detecting M2-1 almost exclusively within inclusion bodies, and the anti-M2-1 antibody detecting it almost exclusively as a diffuse cytoplasmic distribution (Fig 10A and 10C). These findings indicate that different M2-1 epitopes are differentially detected in inclusion bodies versus the cytosol. The 22k4 and 37M2 antibodies used for the CLIP-seq analysis and subsequent validation experiment detected both inclusion body and cytoplasmic distributions of M2-1 (S4 Fig).

M2-1 is phosphorylated on serines S58 and S61 and cycles between a phosphorylated and unphosphorylated state, with both forms being present in infected cells [32,36,57–59]. The phosphorylated form of M2-1 has an ~2-fold lower affinity for RNA oligonucleotides than the unphosphorylated form and does not localize with RSV RNAs in IBAGS [34,58]. While the anti-HA antibody should be able to bind to both the phosphorylated and unphosphorylated forms of M2-1 (provided that the HA epitope was exposed), it was possible that the commercial anti-M2-1 antibody was specific for one form or the other and that a different form of M2-1 accumulates in inclusion bodies versus the cytoplasm. To test this, we performed Western blot analysis of RSV infected cell extracts. The blots were probed with the commercial anti-M2-1 and anti-HA antibodies, used for the immunofluorescence analysis, and the 22k and 37M2 antibodies, used for the CLIP-seq and RNA binding validation experiments. This analysis revealed that all four antibodies detected both forms of the protein, with the unphosphorylated form being detected more prominently under the conditions used (S4 Fig). Therefore, M2-1 phosphorylation status is unlikely to be the explanation for the differential antibody binding that was observed. The reason for the differential staining could be due to different epitopes of M2-1 being exposed in different subcellular locations.

## Discussion

The goal of this study was to identify if M2-1 associates with specific RNA sequences during RSV infection. By using a CLIP-seq approach, it was possible to perform an unbiased analysis and to analyze M2-1: RNA interactions that are occurring in a population of infected cells at early and late times post infection. Our analysis clearly showed that M2-1 binds to positive sense RSV RNAs, but comes into contact with the entire lengths of mRNAs with no sequence specificity. Surprisingly, we observed that M2-1 also bound to a subpopulation of human mRNAs. To our knowledge, this is the first time that evidence for M2-1 binding host mRNA has been presented. In contrast to the non-specific interactions observed with RSV mRNAs, we found that M2-1 binds to specific sites within cellular mRNAs with a preference for AU rich sequences.

M2-1 bound selectively to positive, rather than negative sense, RSV RNAs at both 8 and 18 hpi (Fig 2). It is not possible to distinguish between RSV antigenome and mRNA in this analysis, but given that mRNA is significantly more abundant than antigenome in infected cells [23,60] and M2-1 did not associate with genome RNA, we conclude that it interacts with RSV mRNAs. This result is consistent with previously published findings based on M2-1 immunoprecipitation of radiolabeled RNAs from RSV infected cells that indicated that M2-1 preferentially associated with RSV mRNAs rather than genome RNA, although the results from that study were not quantified and low levels of genome RNA were detectable [32]. This finding suggests that M2-1 functions as an elongation factor by binding to the mRNA transcript, rather than binding to the template, as suggested previously [38].

We found no evidence that M2-1 binds to specific RSV RNA sequences. This was shown with analysis of read density within each of the RSV genes, density of cross-link sites along the RSV transcriptome, and the nucleobase profile adjacent to the cross-link sites (Figs 3–6 and S2). In each of these analyses, the profile of RNA enriched by M2-1 immunoprecipitation was equivalent to that of the SMInput control, with any differences found to be not statistically significant. This indicates that M2-1 contacts the entire RSV transcriptome with no clear bias towards any particular sequence. One possibility that we considered is that the lack of a nuclease digestion step in the CLIP-seq procedure meant that potential binding sites were not clearly defined in the analysis. For example, if the RNA lengths were too long, read ends could occur due to the presence of other RNA binding proteins on the same fragment of RNA. While it is likely that the lack of nuclease digestion did lead to a greater spread of read end sites, it should be noted that the RNA lengths were size selected to be similar to those that would be selected following nuclease digest, and more importantly, there was clearly a difference in the M2-1 binding profiles on RSV RNAs compared to cellular RNAs, in which binding sites were clearly defined (Figs 5 and 9). Although there was no significant difference between the SMInput and IP read profiles, analysis of the RPKM values of the different RSV mRNAs in the SMInput and IP samples revealed that there was a difference in the relative representation of each mRNA, with the RPKM values in the M2-1 IP samples showing a greater trend towards a transcription gradient than the SMInput samples. This was particularly noticeable in a sample that underwent limited nuclease digestion, which would be expected to lead to increased release of nucleocapsids, and from an early timepoint, in which the proportion of nascent RSV mRNAs would have been relatively high (Fig 7A). The trend towards a transcription gradient in the IP samples suggests that M2-1 contacts RSV mRNAs as part of the elongating transcription complex. These data are consistent with minigenome studies of M2-1 function, which have shown that in the absence of M2-1, transcripts of heterogeneous length are generated with no obvious specific termination sites [27,30]. This indicates that in the absence of M2-1, the polymerase is prone to premature transcript release in a stochastic manner whereas in the presence of M2-1 it is able to elongate. Therefore, we propose that M2-1 is a stable component of the transcribing polymerase, contacting the newly synthesized RNA as it exits the product channel of the polymerase, and melting secondary structures that form [61] so that they do not destabilize the polymerase.

Although the data indicate that M2-1 is a component of the transcribing polymerase, with a transcription gradient trend in the IP samples relative to the SMInput samples, the *P* and *F* genes were outliers in every sample. This was most noticeable in the 18 hpi replicate 2 and 3 samples (Figs 7B and S3). This could either be because RSV genes are not always sequentially transcribed in a polar manner, as has been indicated by a study of RSV mRNA abundance and stability [62], or because M2-1 was able to distribute randomly on mRNAs after their release from the polymerase, such that the outlier RPKM values for these two genes partially represent RNA turnover. It is possible that M2-1 is at a very high concentration in inclusion bodies, and particularly in the IBAGS, allowing it to overcome differential affinities for different RNA sequences and bind in a distributive random manner to viral mRNAs that have been released from the polymerase complex.

Previous studies indicated that that M2-1 has high affinity for A-rich sequences such as the gene end sequence or poly(A) tail, and it was suggested that M2-1 might stimulate polyadenylation [34,37,38]. However, the polymerase is capable of generating monocistronic, polyadenylated and translatable mRNAs in the absence of M2-1 [27–29], indicating that M2-1 is not required for gene end signal recognition or polyadenylation. In terms of the CLIP-seq analysis presented here, there was no evidence for preferential binding to gene end sequences (Fig 6). Unfortunately, it is not possible to mine the data to determine if M2-1 exhibits preferential binding to RSV poly A tails compared to the gene end or other RSV sequences for several reasons. First, UV cross-linking occurs more efficiently on uridine residues than other residues and so there would be a bias against detection of homopolymeric A tracts compared to sequences containing a U residue. Second, the poly A tails are variable in length and so how well poly A tracts would be represented in the IP fraction would depend on whether the poly A tail is coated with multiple copies of M2-1 or bound to a single copy. Third, it is not possible to distinguish between poly A sequences of the ten different RSV genes, or between viral and cellular poly A sequences. For these reasons, it is not possible to perform a meaningful comparison of fold-enrichment of RSV poly A sequences versus other RSV sequences, following M2-1 immunoprecipitation. However, a calculation of the percentage of IP reads containing a poly A tract of 20 nt or more (allowing for 1 mismatch per 10 nucleotides) ranged from 0.04 to 0.14% in the different samples (note that this analysis was performed on sequence data in which a step of the data processing pipeline that would normally remove polymeric A tracts was omitted). This leads us to conclude that mRNA poly A tails represented a small proportion of M2-1 bound sequences.

M2-1 bound to human mRNA in addition to RSV mRNA (Figs 2, 8, and 9 and Table 1). At late times in infections, M2-1 is relatively abundant in the cell and it is conceivable that it simply had chance interactions with abundant cellular RNAs. However, in many instances, the M2-1 binding peaks identified in the CLIPper analysis were the same or directly adjacent to each other in multiple replicates, indicating that in these cases, M2-1 was associated with a specific region of the RNA rather than randomly interacting with highly expressed RNAs (Table 1). In addition, specific M2-1 binding to seven of the mRNAs identified by CLIPper analysis, but not *DYNC1H1*, was confirmed by RT-qPCR analysis of M2-1 bound RNA (Fig 8C). This finding indicates that these mRNAs were not false-positive hits generated by the CLIPper analysis, and add further support to the argument that M2-1 is not simply binding to highly abundant mRNAs. In addition, there was considerable overlap between the cellular mRNAs identified at 8 hpi (when M2-1 levels are very low) and 18 hpi. Thus, the data support the conclusion that M2-1 binds specifically to subset of cellular mRNAs. Analysis using motif searching algorithms failed to identify a consensus binding sequence. Failure to identify a specific protein binding motif in CLIP-seq datasets is not unusual [63,64] for numerous reasons. For example, it is possible that M2-1 was associated with RNA as part of a heteromeric protein complex and that the M2-1 binding site is not discernable due to cross-links being introduced between RNA and other proteins within the complex that are adjacent to the M2-1 binding site. Alternatively, it is possible that M2-1 bound two disparate sites on the same RNA molecule such as a secondary structure, or that it has affinity for particular RNA structures, rather than primary sequence. Finally, the bias of UV cross-linking at uridine residues can obscure true binding sites. Although M2-1 was found to have no specific binding interactions with RSV sequences, the analysis of nucleobase composition at the cross-link sites of cellular RNAs showed an enrichment of A and U residues (Fig 5B). Thus, the data regarding cellular RNAs correlate with the findings from biochemical measurements that demonstrated that M2-1 has a high affinity for AU rich oligonucleotides, and with structural data that indicates a specific interaction with an adenosine residue [33,34,37,61]. Binding to specific cellular mRNAs could occur if the concentration of M2-1 in the cytosol is relatively low (as compared to inclusion bodies), such that affinity for specific sequences can come into play.

As yet, the significance of M2-1 binding to cellular RNAs is not known, but there is information from related viruses to suggest that it is important. HMPV M2-1 protein shares significant sequence (38%) and structural homology to its RSV counterpart [44]. Both the RSV and HMPV proteins have a similar domain structure, containing an N-terminal CCCH zinc-finger domain, tetramerization domain, and core domain. In both viruses, the zinc-finger domain can form base-stacking interactions with adenosine, with the core domain having non-specific binding activity [33,34,44]. Whereas RSV M2-1 is required for transcription elongation and for virus viability, HMPV M2-1 is not required for transcription and recombinant virus in which M2-1 is deleted replicates efficiently in cell culture [43,65]. However, HMPV M2-1 is required for infectivity *in vivo* and moreover, this phenotype is dependent on its zinc-finger domain [43,66]. This indicates that in the case of HMPV, the major role of M2-1 is to enable the virus to overcome a barrier that is present *in vivo*, but not in cell culture, through an RNA binding activity. Filoviruses have a structural counterpart to M2-1 called VP30, which is also a CCCH zinc finger protein [67,68]. In the case of EBOV, VP30 serves as an essential transcription factor [69]. It does not have a clear role in MARV transcription [68,70], but is required for recombinant MARV virus replication [71], indicating that VP30 might have an additional role during infection that is unrelated to transcription. The fact that the RSV M2-1 protein is differentially detected by different antibodies (Fig 10) could suggest that it adopts different conformations, allowing it to fulfill two functions, although further experiments are required to determine if this is the case. Cellular CCCH zinc finger proteins are a class of proteins typically involved in post-transcriptional regulation of mRNAs involved in immune responses. They can bind to AU rich sequences (consistent with the cellular RNA binding properties of M2-1) and play a key role in immune regulation, for example by promoting degradation of cytokine mRNAs [72]. It is noteworthy that a number of the target genes identified here are involved in immune responses. For example, CXCL5 is a cytokine involved in neutrophil recruitment [73] and *CANX* codes for calnexin, an endoplasmic reticulum resident protein involved in MHC class I folding [74]. Intriguingly, ZFP36L1 is itself a CCCH zinc finger protein involved in immunomodulation [72]. This raises the possibility that M2-1 functions to regulate immune responses to RSV. Intriguingly both RSV M2-1 and EBOV VP30 proteins have been shown to bind to cellular proteins involved in mRNA metabolism [45,75]. The data presented here suggest the tantalizing possibility that M2-1 and VP30 proteins associate with cellular RNA processing proteins in association with cellular mRNAs to elicit effects on cellular gene expression.

## Materials and methods

### Cells and hybridomas

All cell lines were grown at 37°C with 5% CO_2_. HEp-2 cells (ATCC CCL-23) were cultured in Opti-MEM (Gibco) supplemented with 2% FBS (Gibco) and 1% GlutaMAX Supplement (Gibco). A549 cells (ATCC CCL-185) were grown in DMEM supplemented with 10% fetal bovine serum (FBS) and 1% Antibiotic-Antimycotic Solution (Gibco) or 1% Penicillin-Streptomycin (Gibco). Cells were passaged using 0.25% Trypsin-EDTA (Gibco). FBS used for cell culture was heat-inactivated for 30 minutes at 56°C. Hybridomas that produce 22k4 and 37M2 antibodies against M2-1 were a gift from Dr. José Melero, (Instituto de Salud Carlos III) [49], and grown in ClonaCell-HY Medium E (StemCell Technologies). All cells were routinely mycoplasma tested with either the EZ-PCR Mycoplasma Detection Kit (Biological Industries) or Maxim Biotech MPCR Kit for Mycoplasma Detection (Fisher Scientific).

### Viruses

The human RSV A2 stocks used for the CLIP-Seq analysis and immunofluorescence studies were generated from a virus stock received as a gift from Dr. Peter Collins (NIAID, NIH). The human RSV A2 stock used for the RT-qPCR analysis was obtained from ATCC (VR-1544). To generate the HA-tagged M2-1 recombinant virus, rRSV_HA-M2-1_, a plasmid that encodes the RSV antigenome was used. This cDNA contains a mutation in the SH gene that does not affect the protein sequence, but does increase plasmid stability. The plasmid also contains an introduced BsiWI site in the trailer region [60]. To facilitate the insertion of HA at the N terminus of the M2-1 protein, M2 sequence located within unique restrictions sites SacI and BamHI was cloned into a modified pGEM T easy vector (Promega) to generate a shuttle vector. Next, the HA tag sequence was inserted at the N terminus of M2-1 using a Q5 Site-Directed Mutagenesis kit (NEB) and primers 5’ GAT GTT CCA GAT TAC GCT TCA CGA AGG AAT CCT TGC and 5’ GTA TGG GTA **CAT** *ATT TGC CCC* AGT TTT CAT TTT TAC AG, in which the HA specific sequence is underlined, the start codon sequence is in bold type and the gene start signal for the M2 gene is italicized. The HA-M2-1 sequence in the shuttle vector was subsequently cloned into the full-length RSV antigenome construct using the SacI and BamHI restriction sites. Full-length clones of RSV encoding a HA-tagged M2-1 were subjected to restriction digests and sequencing to confirm their identity. The virus was rescued as previously described and sequenced to confirm the presence of the tag. To propagate RSV, cells at 80% confluence were washed with PBS without Ca^2+^ and Mg^+^ (Lonza) and virus was added at a multiplicity of infection of 0.1 plaque forming units/ cell diluted in complete medium for 1 h at 37°C. Complete medium was then added to the culture for prolonged inoculation at 37°C. When high cytopathic effects were observed (~4–7 days post infection), cells were scraped into the medium, cell debris was separated, and supernatant was aliquoted and stored at -80°C. Virus titers were determined via plaque assay as previously described [60]. All viruses, including rRSV_HA-M2-1_, reached titers of 10^7^ pfu/mL.

### Optimization of conditions for seCLIP-seq analysis

To confirm the conditions used selectively immunoprecipitated M2-1 in the absence of other RSV proteins, A549 cells in 10 cm dishes were infected with RSV A2 at an moi of 3 pfu/cell. At 18 hpi, cells were exposed to UV light at 40 mJ/cm^2^. Cells were lysed in 1 mL of RIPA buffer (50 mM Tris-HCL (pH 7.4), 150 mM NaCl, 1% NP40, 0.5% NaDOC, 0.1% SDS) and sonicated for 5 cycles of 30 seconds on/30 seconds off at 4°C in a Bioruptor Pico Sonicator (Diagenode). Lysate was incubated with 2 μL of Turbo DNase (Ambion) with varying concentrations of RNase I (see S2 Fig) then incubated at 1,200 rpm/37°C/5 min in a thermomixer. Lysate was immediately placed on ice and 11 μL of murine RNase Inhibitor (NEB) was added. Lysate was spun at 15,000 x g at 4°C for 15 minutes and supernatant was transferred to a new tube. Lysates were pre-cleared with 10 μL of washed Dynabeads Protein G (Invitrogen) for 45 minutes at 4°C. 20 μL of antibody 22k4 was coupled to 100 μL of beads for 1 hr at RT. Lysate was incubated with antibody-coupled beads overnight at 4°C. Beads were washed in a 1:1 mix of RIPA buffer and high salt wash buffer (50 mM Tris-HCL (pH 7.4), 1 M NaCl,1 mM EDTA 1% NP40, 0.5% NaDOC, 0.1% SDS), twice with high salt wash buffer, then a 1:1 mix of high salt wash buffer and wash buffer (20 mM Tris-HCL (pH 7.4), 10 mM MgCl_2_, 0.2% Tween-20), then once with wash buffer. 20 μL of beads were removed and combined with 7.5 μL of 5x loading dye and 3 μL of 1 M DTT, incubated at 95°C for 5 minutes/1,200 rpm and migrated on a 12% SDS-PAGE gel. Western blot analysis was performed as described above, with a 1:1000 dilution of primary M2-1 and RSV antibodies and a 1:20,000 dilution of Li-Cor secondary antibodies.

To confirm that conditions used selected for RNA cross-linked to protein, A549 cells were mock infected or infected with RSV A2 and exposed to UV light at 40 mJ/cm^2^ or not crosslinked before lysis. M2-1 was immunoprecipitated with 22k4 antibody and washed as described above. Beads were resuspended in 50 μL of 1X polynucleotide kinase buffer (NEB) with 20 μCi of [γ -^32^P] ATP and 20 units of T4 PNK (NEB). Reactions were incubated for 1 h at 1,000 rpm/37°C. Reactions were stopped by adding 11 μL of 4x loading dye and 5 μL of 1M DTT. Reactions were loaded on a 12% NuPAGE gel (Invitrogen), migrated, and then transferred to nitrocellulose as previously described. The nitrocellulose was then analyzed by autoradiography.

### Western blot analysis

Cells were resuspended in either NP40 or RIPA lysis buffer and lysates were combined with 50 mM DTT and Novex Tris-Glycine SDS Sample Buffer to a final dilution of 1X. Samples were heated at 95°C for 5 minutes and migrated on a tris-glycine or NuPAGE gel (Invitrogen). Protein was transferred to a nitrocellulose membrane using XCell II blot module (Invitrogen) as per manufacturer’s instructions. Blots were analyzed with the primary antibodies HA.11 (Biolegend, 901502), αM2-1 (Abcam, ab94805), 22k4, and 37M2, and αRSV (Abcam, ab20745) at a dilution of 1:500–1:1000 and LI-COR secondary antibodies (Li-Cor, 925–68072, 925–68073, and 925–32214) in a dilution of 1:20,000 in PBST. Membranes were imaged on the LI-COR Odyssey. The method for the Western blots shown in S4 Fig were similar, except that cells were lysed directly in SDS-PAGE lysis buffer and lysates were subjected to centrifugation through a Qiashredder column.

### seCLIP-seq analysis

The seCLIP procedure was performed according to the protocol described by Van Nostrand and coworkers [48], with some modifications. A549 cells in 10 cm dishes were infected with RSV A2 at an moi of 3 pfu/cell or mock infected. At 8 or 18 hpi, cells were exposed to UV light and lysed as previously described with the exception of 8 and 18 hpi replicate 1 samples in which RNase inhibitor was omitted, allowing for limited RNAse digestion by cellular nucleases. Lysate was spun at 15,000 x g at 4°C for 15 minutes and supernatant was transferred to a new tube. M2-1 was immunoprecipitated as described above with the exception that immediately after overnight incubation with 22k4 antibody, 2% of the lysate:bead mix was removed and reserved as the SMInput sample. The remaining beads were washed as described above and incubated at 1,200 rpm at 37°C for 15 minutes. IP RNA samples were then dephosphorylated with FastAP (Life Technologies) then phosphorylated while shaking with T7 polynucleotide kinase (NEB) at 1,200 rpm at 37°C for 20 minutes in the thermomixer. Primer InvRiL19 (S2 Table) was ligated onto the 3´ end of the RNA with T4 RNA ligase (NEB). Samples were migrated alongside SMInput RNA on a 12% NuPAGE SDS-PAGE gel and transferred to nitrocellulose. A region corresponding to the protein of interest crosslinked to 50–300 nt of RNA was excised from the gel. RNA was released from the bound protein and membrane by digestion with proteinase K (NEB) for 20 min/37°C/1,200 rpm followed addition of proteinase K buffer with 7M urea for 20 min/ 37°C/ 1,200 rpm. RNA was then purified with acid phenol/chloroform/isoamyl alcohol (pH 4.5) and the aqueous layer was combined with 100% ethanol, and collected with the RNA Clean & Concentrator-5 (Zymo Research). RNA from the SMInput was dephosphorylated and then phosphorylated as described above then ligated to InvRiL19 and purified on MyOne Silane Dynabeads (Invitrogen). Both M2-1 immunoprecipitated and SMInput samples were reverse transcribed with Affinity Script reverse transcriptase (Agilent) with primer InvAR17 and nucleic acids were isolated using an ExoSAP-IT kit (ThermoFisher Scientific). RNA was removed by NaOH cleavage and cDNA was purified on MyOne Silane Dynabeads (Invitrogen) and then eluted in 10 mM Tris-HCL pH 7.5. Primer InvRand3Tr3 was ligated to cDNA samples with T4 RNA ligase (NEB) and incubated overnight at room temperature. DNA samples were bound to MyOne Silane Dynabead, washed, and eluted before individual cDNA libraries were generated for each sample by PCR using barcoded primers to allow different samples to be distinguished following sequencing. PCR products were purified using AmpureXP beads (Beckman) and migrated on a 3% low-melting temperature agarose gel. Slices were cut from the gel and DNA was extracted with the MinElute Gel Extraction Kit (Qiagen) according to manufacturer’s instructions. Samples were combined into one library, which was evaluated for quality on a stand analyzer and subjected to single end, 50 nt sequencing on Illumina’s HiSeq2500 platform.

### Computational analysis of seCLIP-seq data

The full details of the computational pipeline are in S1 Method. Briefly, low quality sequences were discarded and Illumina adapter sequences were trimmed using Trimmomatic [76]. Unique molecular identifiers (UMI) were extracted from reads and moved to the read title using UMI-tools [77]. Repetitive elements were removed and sequences were aligned to the human (hg19) and RSV (NCBI Accession # M74568.1) genomes using STAR aligner [78]. Reads were sorted and indexed using SAM tools [79] and PCR duplicates were removed with UMI-tools. To analyze the nucleotide resolution of M2-1 binding to RSV mRNA, reads that aligned to positive sense RSV RNA were separated and the number of read-start occurrences per position was divided by the average number of read-starts corresponding to the RSV genome in each sample. These data were visualized using R/ggplot2. T-tests were performed at each position by comparing the mean of the IP replicates to the mean of the SMInput replicates for either 8 or 18 hpi. P-values were adjusted for multiple t-tests, controlling for the false discovery rate. The peak-finding program, CLIPper, was used to find significant peaks in hg19-aligned reads [52]. BEDTools was used to find overlapping peaks from CLIPper analysis and to assign gene identities to each peak [80]. U-enrichment plots were generated by determining the nucleotide sequence flanking each read end using a script from the laboratory of Jernej Ule (https://github.com/ulelab/non-coinciding_cDNA_starts/blob/master/Other_scripts/flankBEDpositions.py) [51], calculating the nucleotide content using HOMER, then graphing results with ggplot2. The sequence data have been deposited to the NCBI Sequence Read Archive (BioProject: PRJNA655902). Poly A content was evaluated using Cutadapt, searching for a string of 20 “A” residues, allowing for 1 error for every 10 nucleotides.

### Confirmation of RNA binding to M2-1 using RT-qPCR

A549 cells were infected with RSV-A2 at an moi of 3 pfu/cell and incubated at 37°C for 18 hours. Cells were washed with PBS and then cells were subjected to UV light at 40 mJ/cm^2^. Cells were scraped into PBS and collected by centrifugation at 200 x g for 5 minutes at 4°C. Cells were lysed in 1 mL of RIPA buffer and incubated on ice for 15 minutes followed by treatment with 2 μL of Turbo DNAse (Ambion) in a thermomixer at 1,200 rpm for 5 minutes at 37°C. Cell lysates were supplemented with 11 μL of murine RNase inhibitor and subjected to centrifugation at 15,000 x g for 15 minutes at 4°C. The supernatant was precleared by addition to beads and then collected. Half the supernatant was reserved as an unenriched control and the remainder was incubated with beads bound to antibody 37M2 for at 12–15 h at 4°C with rotation. Beads were washed as described above. Following removal of the last wash, beads were incubated with proteinase K, then a mixture of proteinase K buffer and 7 M urea as described above. Following protein digest, RNA was purified by phenol:chloroform extraction and ethanol precipitation as described above.

250 ng of immunoprecipitated RNA and 1,000 ng of no-immunoprecipitation RNA was reverse transcribed using SuperScript IV reverse transcriptase. RNA was combined with random hexamers (Invitrogen) and dNTPs per manufacturer’s instructions, heated at 65°C for 5 minutes and incubated on ice for 1 min. Primer:RNA mix was combined with 1X SuperScript IV reverse transcriptase buffer (Invitrogen), DTT, RNase Inhibitor, and 100 U of SuperScript IV reverse transcriptase (Invitrogen). The reaction mix was incubated at room temperature (23°C) for 10 minutes, incubated at 55°C for 20 minutes and heat inactivated at 80°C for 10 minutes. The cDNA was then combined with iTaq universal SYBR green supermix, 200 nM primers, and water added to a final volume of 10 μL, where the cDNA represented 10% of the final qPCR mix. qPCR mixes were prepared in 96-well low-profile, unskirted multiplates (Bio-Rad) and sealed with TempPlate RT optically clear film. qPCR was then performed using the Bio-Rad CFX90 Real-Time PCR platform with the following protocol: (1) 95°C for 30 seconds (2) 95°C for 5 seconds (3) 60°C for 25 seconds (4) repeat steps 2 and 3 39 times (5) Melt Curve analysis. Fold change over N was calculated by subtracting the C(t)_IP_ from the C(t)_unenriched_ samples, then dividing that by C(t)_RSV N- unenriched_—C(t) _RSV N-IP_. Primers for qPCR are detailed in S3 Table. The specificities of all primers used were confirmed prior to use by performing RT-PCR reactions and analyzing products on an agarose gel and determining their size alongside molecular weight standards.

### Immunofluorescence microscopy

For analysis of rRSV_HA-M2-1_ virus, A549 cells were seeded in a 96 well glass bottom plate (Cell-Vis), infected or mock infected with rRSV_HA-M2-1_ at an moi of 3 pfu/ cell and fixed at indicated timepoints with 4% paraformaldehyde for 10 minutes at room temperature. Cells were then permeabilized with 0.2% Triton X-100 (Sigma Aldrich) for 5 minutes at room temperature and blocked with 5% BSA for 30 minutes at 37°C before being incubated with primary antibodies specific to M2-1 (Abcam, ab94805) and HA (Y-11) (Santa Cruz, sc-805) for 30 minutes at 37°C. Cells were then washed with PBS and incubated with AlexaFluor 488 (Thermofisher, A-21202) and AlexaFluor 546 (Thermofisher, A10040) secondary antibodies for 30 minutes at 37°C. Cell nuclei were stained with 4’,6-diamidino-2-phenylindole (DAPI) (Life Technologies), and Prolong Gold (Life Technologies) was added on top of cells before imaging using a 63x, NA 1.4 Zeiss Plan-Apochromat oil objective with a Hamamatsu Flash 4.0 v2 sCMOS camera on a PerkinElmer UltraView spinning disk confocal microscope on a Zeiss Axiovert 200M body. Volocity acquisition software (Perkin Elmer) was utilized to record image stacks at 200 nm intervals. Images were linearly contrast enhanced for clarity.

For immunofluorescence analysis with 37M2 and 22k4 antibodies, A549 cells were seeded on glass coverslips in a 12-well plate and were either mock infected or infected with RSV A2 at an moi of 1 pfu/cell. At 8 or 18 hpi post infection, cells were fixed with 5% formaldehyde with 2% sucrose in PBS for 30 minutes at room temperature and permeabilized with 0.5% IGEPAL with 10% sucrose in PBS for 20 minutes at 4°C. Cells were blocked in PBS with 2% FBS and stained with primary antibody diluted in blocking buffer overnight at 4°C. After washing with 1X PBS, cells were stained with secondary antibodies labeled with AlexaFluor 488 and 4’,6-diamidino-2-phenylindole (DAPI) in a final concentration of 2 ng/μL. Cells were washed with PBS and mounted with mounting media, cell side down. Cover slides were sealed with nail polish and stored at 4°C prior to fluorescence microscopy.

## Supporting information

S1 Fig Optimization experiments to reduce co-precipitation of RSV nucleocapsid associated proteins. Western blot analysis to examine specificity of M2-1 immunoprecipitation under different UV irradiation and RNase I digest conditions. A549 cells in 10 cm dishes were infected with RSV A2 at an moi of 3 pfu/cell. At 18 hpi, cells were exposed to UV-C light at 40 or 400 mJ/cm^2^. Cells were lysed, sonicated, and digested with DNase (Ambion) as described in the main text with the addition of the indicated amounts of RNase I (Ambion). Samples were then incubated at 1,200 rpm/37°C/5 min in a thermomixer. Lysate was immediately placed on ice and murine RNase Inhibitor (NEB) was added and insoluble portion was removed as described above. Supernatants were immunoprecipitated for M2-1 as described in the main text, with the exception that antibody 37M2 was used. Beads were resuspended in 50 μL of RIPA buffer + 12.5 μL of 5x loading dye and 5 μL of 1 M DTT, incubated at 95°C for 5 minutes/1,200 rpm and migrated on a 12% SDS-PAGE gel. Western blot analysis was performed as described in the main text with a 1:1000 dilution of primary antibody αRSV (Abcam ab20745) and a 1:20,000 dilution of secondary antibody IRDye 800CW Donkey anti-goat IgG (LI-COR, 925–32214). Co-purifying RSV proteins are labeled. Heavy and light chains from the antibody used for immunoprecipitation (37M2 antibody) are seen on the Western blot.
(TIF)

S2 Fig Read coverage of the RSV genome in SMInput and IP samples at 8 hpi. Coverage tracks of RSV positive sense (predominately mRNA) reads for each of the individual replicates at 8 and 18 hpi (A and B, respectively), with the tracks for the IP and SMInput samples shown in blue and orange, respectively. Images were generated using IGV. Note that the *y*-axes are scaled differently for each track to allow direct comparison.
(TIF)

S3 Fig M2-1: RNA interactions show evidence of a transcription gradient. RPKM values were calculated for RSV positive sense sequences represented in the SMInput and IP datasets and plotted against each of the RSV genes. The figure shows graphs for each of the samples: 8 hpi replicate 1 (A), 8 hpi replicate 2 (B), 18 hpi replicate 1 (C), 18 hpi replicate 2 (D), 18 hpi replicate 3 (E).
(TIF)

S4 Fig Characterization of M2-1 specific antibodies. (A) Immunofluorescence of RSV A2 infected A549 cells fixed at 18 hpi, using the indicated antibodies. (B-D) Western blot analysis to examine the sensitivities of M2-1 antibodies to phosphorylated and unphosphorylated M2-1. A549 cells were mock infected, or infected with wt RSV A2 or rRSV_**HA-M2-1**_, as indicated. At 18 hpi, cells were harvested and lysed in 1 X SDS-PAGE buffer, the DNA was sheared using a QiaShredder (Qiagen) and the lysates were subjected to Western blot analysis. The samples were migrated in duplicate on each Western blot and the blot was cut in half following transfer. One half of each blot was probed with the commercial anti-M2-1 antibody, and the other half was probed with anti-HA (B), 22k4 (C) or 37M2 (D) antibodies. In the case of the anti-HA antibody, a longer exposure is shown, in addition, to reveal the very faint band representing phosphorylated M2-1. On each blot, detectable phosphorylated M2-1 is indicated with an asterisk.
(TIF)

S1 Method Data processing pipeline. (DOCX)

S1 Table Read counts for each of the CLIP-seq samples. (DOCX)

S2 Table Primers used for seCLIP-seq. (DOCX)

S3 Table Primers used for RT-qPCR. (DOCX)
