# Supplementary figures and images for: Respiratory syncytial virus M2-1 protein associates non-specifically with viral messenger RNA and with specific cellular messenger RNA transcripts
Source: PLoS Pathog. 2021 May 18;17(5):e1009589. doi: 10.1371/journal.ppat.1009589 (PMC8162694; doi:10.1371/journal.ppat.1009589)

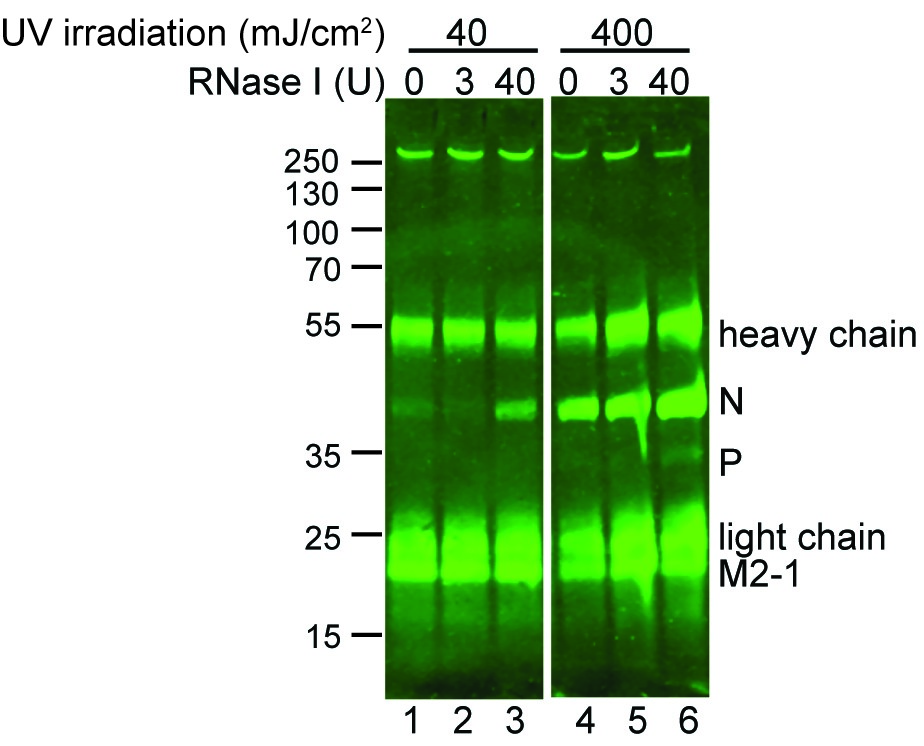

Supplement: S1 Fig — Western blot analysis to examine specificity of M2-1 immunoprecipitation under different UV irradiation and RNase I digest conditions. A549 cells in 10 cm dishes were infected with RSV A2 at an moi of 3 pfu/cell. At 18 hpi, cells were exposed to UV-C light at 40 or 400 mJ/cm2. Cells were lysed, sonicated, and digested with DNase (Ambion) as described in the main text with the addition of the indicated amounts of RNase I (Ambion). Samples were then incubated at 1,200 rpm/37°C/5 min in a thermomixer. Lysate was immediately placed on ice and murine RNase Inhibitor (NEB) was added and insoluble portion was removed as described above. Supernatants were immunoprecipitated for M2-1 as described in the main text, with the exception that antibody 37M2 was used. Beads were resuspended in 50 μL of RIPA buffer + 12.5 μL of 5x loading dye and 5 μL of 1 M DTT, incubated at 95°C for 5 minutes/1,200 rpm and migrated on a 12% SDS-PAGE gel. Western blot analysis was performed as described in the main text with a 1:1000 dilution of primary antibody αRSV (Abcam ab20745) and a 1:20,000 dilution of secondary antibody IRDye 800CW Donkey anti-goat IgG (LI-COR, 925–32214). Co-purifying RSV proteins are labeled. Heavy and light chains from the antibody used for immunoprecipitation (37M2 antibody) are seen on the Western blot. (TIF) [file ppat.1009589.s001.tif]

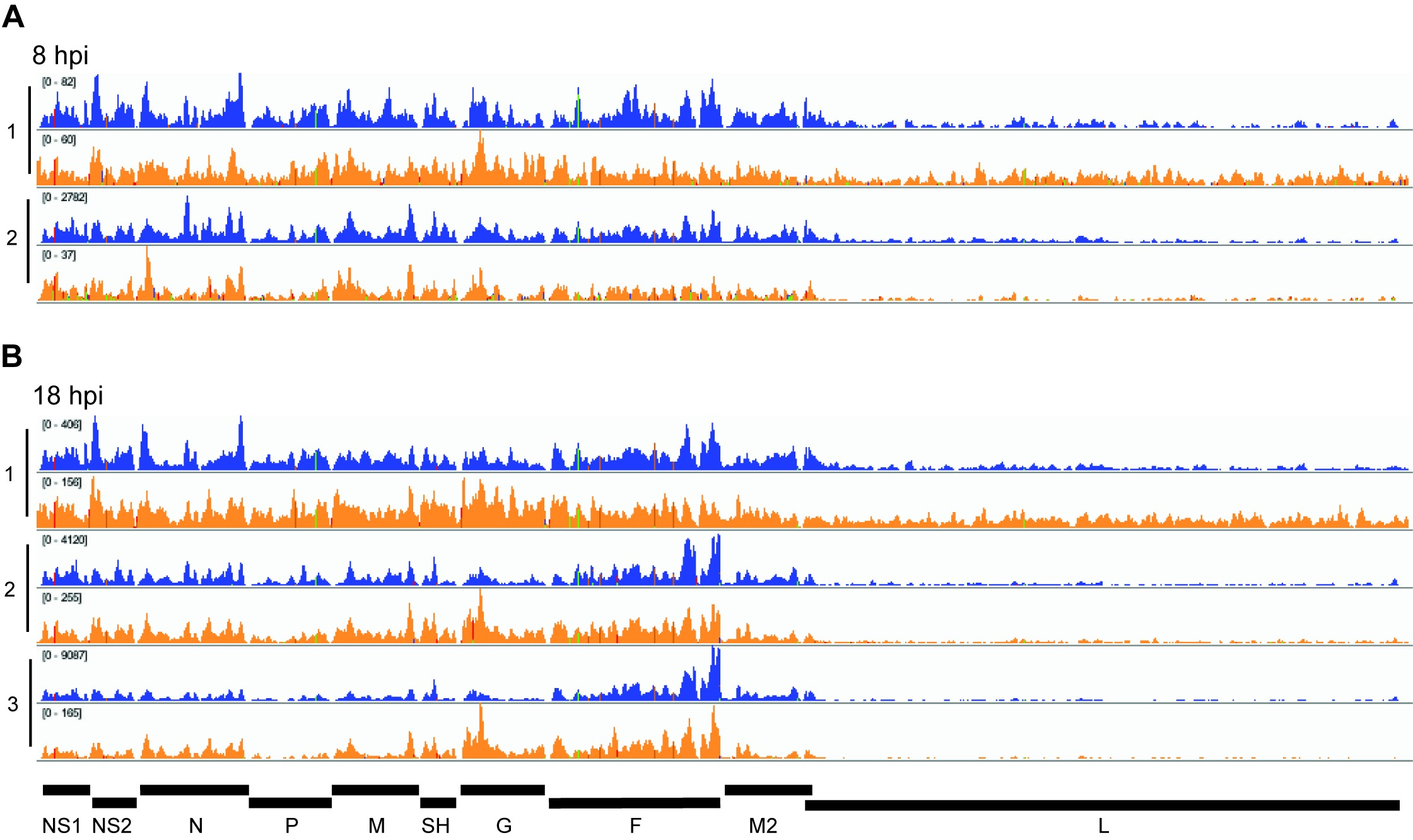

Supplement: S2 Fig — Coverage tracks of RSV positive sense (predominately mRNA) reads for each of the individual replicates at 8 and 18 hpi (A and B, respectively), with the tracks for the IP and SMInput samples shown in blue and orange, respectively. Images were generated using IGV. Note that the y-axes are scaled differently for each track to allow direct comparison. (TIF) [file ppat.1009589.s002.tif]

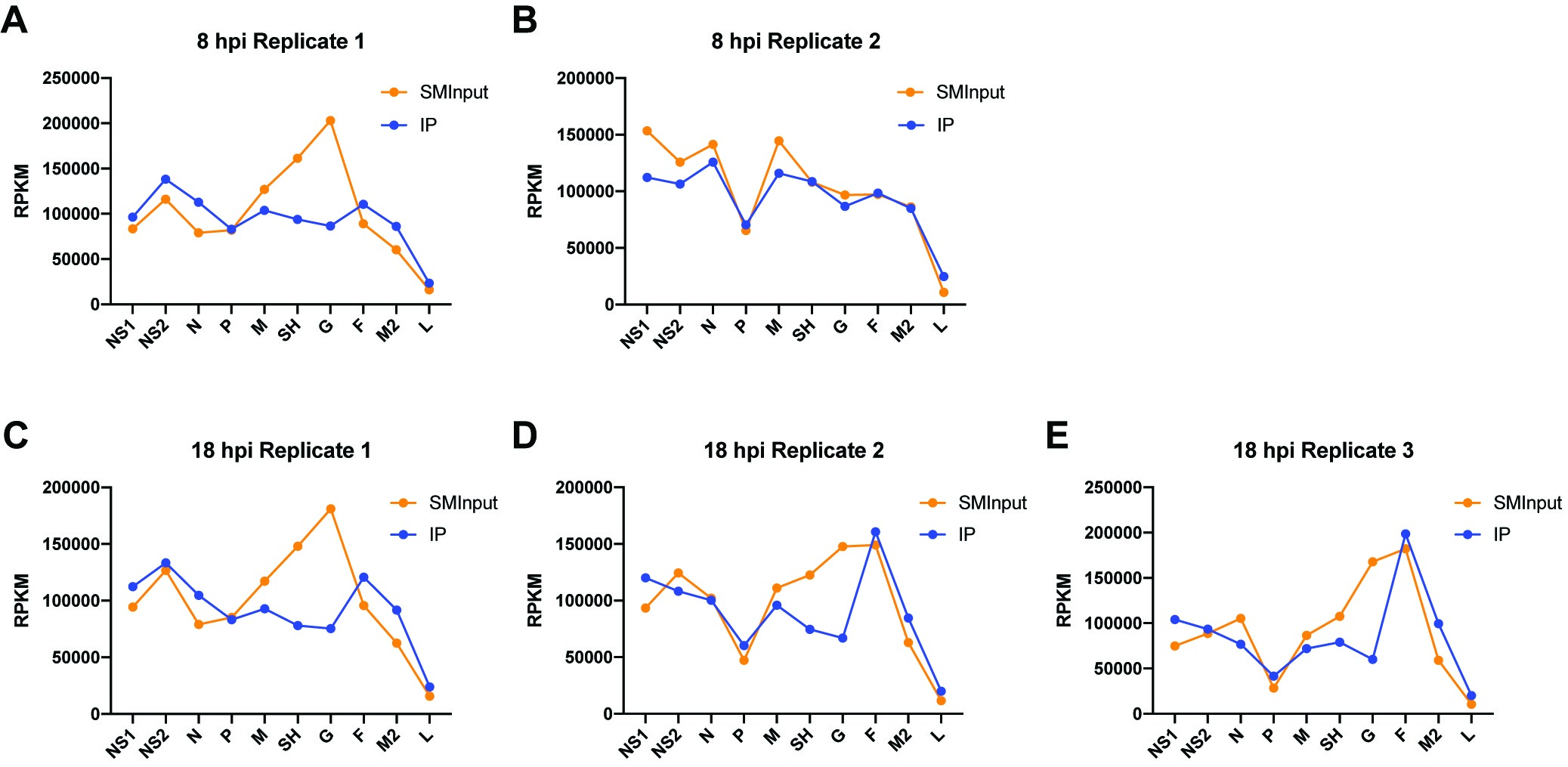

Supplement: S3 Fig — RPKM values were calculated for RSV positive sense sequences represented in the SMInput and IP datasets and plotted against each of the RSV genes. The figure shows graphs for each of the samples: 8 hpi replicate 1 (A), 8 hpi replicate 2 (B), 18 hpi replicate 1 (C), 18 hpi replicate 2 (D), 18 hpi replicate 3 (E). (TIF) [file ppat.1009589.s003.tif]

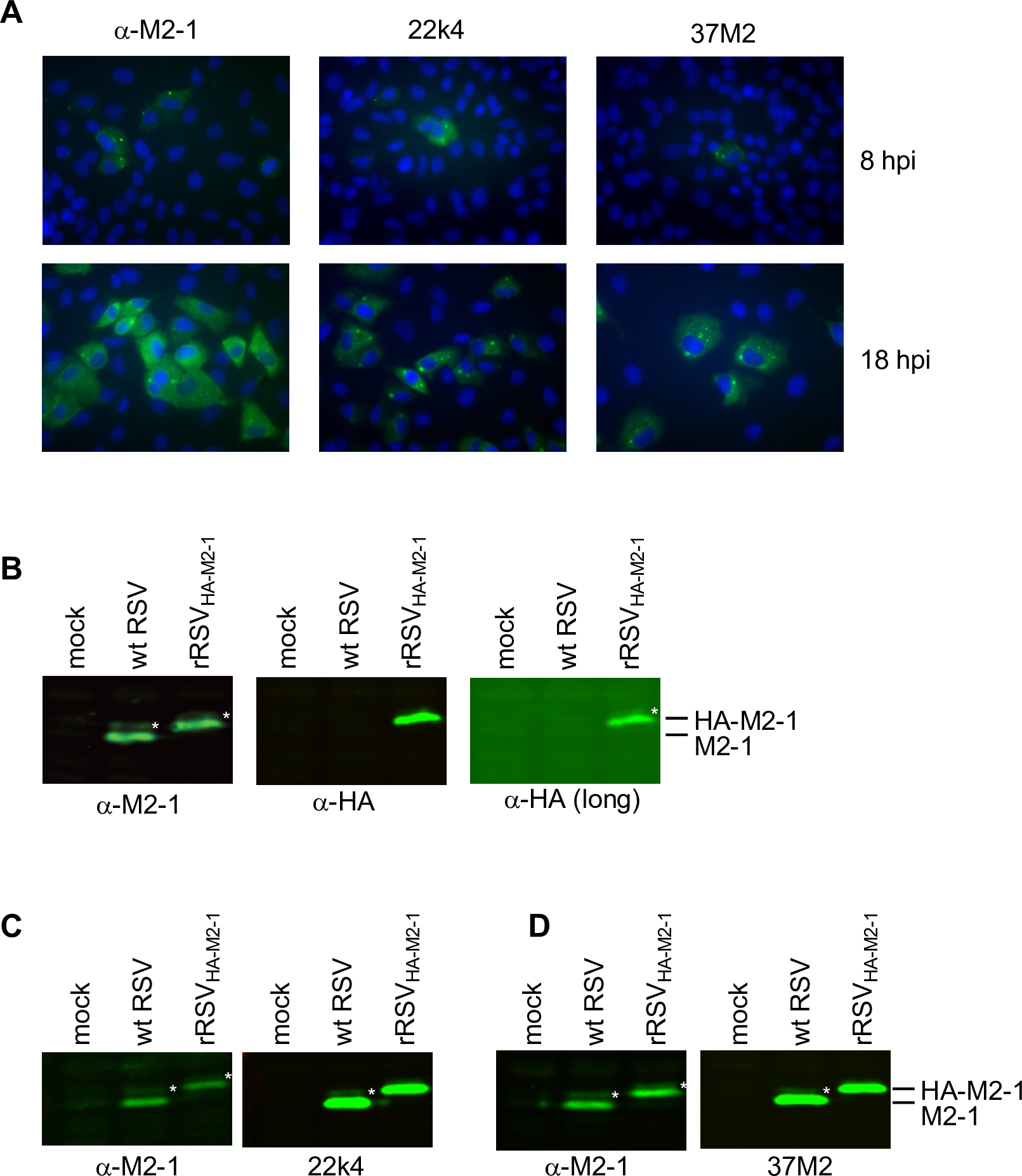

Supplement: S4 Fig — (A) Immunofluorescence of RSV A2 infected A549 cells fixed at 18 hpi, using the indicated antibodies. (B-D) Western blot analysis to examine the sensitivities of M2-1 antibodies to phosphorylated and unphosphorylated M2-1. A549 cells were mock infected, or infected with wt RSV A2 or rRSVHA-M2-1, as indicated. At 18 hpi, cells were harvested and lysed in 1 X SDS-PAGE buffer, the DNA was sheared using a QiaShredder (Qiagen) and the lysates were subjected to Western blot analysis. The samples were migrated in duplicate on each Western blot and the blot was cut in half following transfer. One half of each blot was probed with the commercial anti-M2-1 antibody, and the other half was probed with anti-HA (B), 22k4 (C) or 37M2 (D) antibodies. In the case of the anti-HA antibody, a longer exposure is shown, in addition, to reveal the very faint band representing phosphorylated M2-1. On each blot, detectable phosphorylated M2-1 is indicated with an asterisk. (TIF) [file ppat.1009589.s004.tif]
