# Supplementary material for: Respiratory syncytial virus M2-1 protein associates non-specifically with viral messenger RNA and with specific cellular messenger RNA transcripts
Source: PLoS Pathog. 2021 May 18;17(5):e1009589. doi: 10.1371/journal.ppat.1009589 (PMC8162694; doi:10.1371/journal.ppat.1009589)
Supplement: S1 Method — (DOCX) [file ppat.1009589.s005.docx]

**S1 Method**

**Data processing pipeline**

Align sequences to human and RSV genomes and find Peaks in human alignments

#0. Load all necessary applications

module load python/2.7.13

module load fastqc/0.11.7

module load trimmomatic/0.36

module load umitools/0.5.4

module load samtools/1.8

module load gcc/6.2.0

module load star/2.5.3a

module load clipper/1.2.1

module load bedtools

#1. Fastqc – assess the quality of the reads

fastqc -o /full/path/to/input/files/file.fastq.gz

#2. Trimmomatic TM.fastq.gz – remove low quality and short reads

trimmomatic SE -phred33 /full/path/to/input/files/file.fastq.gz /full/path/to/output/files/file.fastq.gz \

ILLUMINACLIP:"$SCC_TRIMMOMATIC_ADAPTERS"/TruSe3-SE.fa:2:30:10 LEADING:0 TRAILING:3 SLIDINGWINDOW:4:18 MINLEN:30

#3. UMI extract .TM.ext.fastq.gz – move the UMI to the read name line

umi_tools extract --extract-method=string --bc-pattern=NNNNNNNNNN \

-I /full/path/to/input/files/file.TM.fastq.gz -S /full/path/to/output/files/file.TM.ext.fastq.gz -L path/to/log/file_umiextract.log

#4. STAR rmRep .TM.ext.rmRep.bam - remove spurious artifacts from repeats in the genome and rRNA

star --runMode alignReads

--runThreadN 16 --genomeDir /full/path/to/homo_sapiens_repbase --genomeLoad LoadAndRemove --readFilesIn /full/path/to/input/files/file.TM.ext.fastq.gz \

--readFilesCommand zcat --outSAMunmapped Within --outFilterMultimapNmax 30 --outFilterMultimapScoreRange 1 --outFileNamePrefix /full/path/to/output//files/file.TM.ext.rmRep.bam \

--outSAMattributes All --outSAMtype BAM Unsorted --outFilterType BySJout --outReadsUnmapped Fastx --outFilterScoreMin 10 \

--outSAMattrRGline ID:foo --alignEndsType EndToEnd

#5. Fastqc – assess the quality of the reads post clean up

fastqc -o /full/path/to/input/files/file.TM.ext.rmRep.bamUnmapped.out.mate1

#6. Star genome mapping rsv .TM.ext.rmRep.rsv.bam – align the RSV genome

STAR --runMode alignReads --runThreadN 16 --genomeDir path/to/RSV/genome/star_index --genomeLoad LoadAndRemove \

--readFilesIn /full/path/to/input/files/file.TM.ext.rmRep.bamUnmapped.out.mate1 --outSAMunmapped Within --outFilterMultimapNmax 10 --outFilterMultimapScoreRange 1 --alignIntronMax 1 \

--outFileNamePrefix /full/path/to/output/files/file.ext.rmRep.rsv --outSAMattributes All --outSAMtype BAM Unsorted --outReadsUnmapped Fastx --outFilterScoreMin 10 \

--outFilterMismatchNoverReadLmax 0.1 --alignEndsType EndToEnd --quantMode GeneCounts

#7. Star genome mapping hg19 – align to hg19 genome

STAR --runMode alignReads --runThreadN 16 --genomeDir path/to/hg19/genome/star_index --genomeLoad LoadAndRemove \

--readFilesIn /full/path/to/input/files/file.TM.ext.rmRep.bamUnmapped.out.mate1 --outSAMunmapped Within --outFilterMultimapNmax 1 --outFilterMultimapScoreRange 1 \

--outFileNamePrefix /full/path/to/output/files/ file.TM.ext.rmRep.hg19 --outSAMattributes All --outSAMtype BAM Unsorted --outFilterType BySJout --outReadsUnmapped Fastx \

--outFilterScoreMin 10 --outSAMattrRGline ID:foo --alignEndsType EndToEnd --quantMode GeneCounts

#8. Samtools sort TM.ext.rmRep.hg19.bam OR .TM.ext.rmRep.rsv.sort.bam

samtools sort /full/path/to/input/files/file.TM.ext.rmRep.rsvAligned.out.bam -o /full/path/to/output/files/file.TM.ext.rmRep.rsvAligned.sort.bam

samtools sort /full/path/to/input/files/file.TM.ext.rmRep.hg19Aligned.out.bam -o /full/path/to/output/files/file -IP.TM.ext.rmRep.hg19Aligned.sort.bam

#9. Samtools index same file names as #9

samtools index /full/path/to/input/files/file.TM.ext.rmRep.rsvAligned.sort.bam

samtools index /full/path/to/input/files/file.TM.ext.rmRep.hg19Aligned.sort.bam

#10. UMI dedup .TM.ext.rmRep.hg19.sort.rmDup.bam OR .TM.ext.rmRep.rsv.sort.rmDup.bam – de-duplicate PCR reads

umi_tools dedup --method unique -I /full/path/to/input/files/file.TM.ext.rmRep.rsvAligned.sort.bam -S /full/path/to/output/files/file.rsvFinal.bam -L path/to/log/file.rsv

umi_tools dedup --method unique -I /full/path/to/input//files/file.TM.ext.rmRep.hg19Aligned.sort.bam -S /full/path/to/output/files/file.hg19Final.bam -L path/to/log/file.hg19

#11. Samtools index same file names as #10

samtools index /full/path/to/input/files/file.rsvFinal.bam

samtools index /full/path/to/input/files/file.hg19Final.bam

#12. Covert bam to sam file

samtools view -h -o /full/path/to/output/files/file.rsvFinal.sam /full/path/to/input/files/file.rsvFinal.bam

samtools view -h -o /full/path/to/output/files/file.hg19Final.sam /full/path/to/input/files/file.hg19Final.bam

#13. Run CLIPper to determine peaks (for human data only) – finds peaks

clipper -b /full/path/to/input/files/file.hg19Final.bam -s hg19 -o /full/path/to/output/files/file.hg19clip.bam

Fix scores of CLIPper analysis

module load python

module load samtools

module load perl/5.24.0

samtools view -c -F /full/path/to/input/files/file.hg19Final.bam > /full/path/to/output/files/readnum/file.experiment_read_num_file

perl overlap_peakfi_with_bam_PE.pl /full/path/to/input/files/file(IP).hg19Final.bam /full/path/to/input/files/file(SMI).hg19Final.bam full/path/to/input/CLIPper/files/file.hg19clip.bed /full/path/to/input/files/readnum/file(IP).experiment_read_num_file /full/path/to/input/files/readnum/file(SMI).experiment_read_num_file /full/path/to/output/files/file.normalized

perl peakscompress.pl /full/path/to/output/files/file.normalized /full/path/to/output/files/file.single
