# Supplementary material for: Respiratory syncytial virus M2-1 protein associates non-specifically with viral messenger RNA and with specific cellular messenger RNA transcripts
Source: PLoS Pathog. 2021 May 18;17(5):e1009589. doi: 10.1371/journal.ppat.1009589 (PMC8162694; doi:10.1371/journal.ppat.1009589)
Supplement: S1 Table — (DOCX) [file ppat.1009589.s006.docx]

**S1 Table. Read counts for each of the CLIP-seq samples***.* The table shows read counts at different stages of the pipeline for the different samples described in the text. Note that the unmapped reads include unmapped reads, PCR duplicates, rRNA and human genome repeat elements.
