# Supplementary material for: Respiratory syncytial virus M2-1 protein associates non-specifically with viral messenger RNA and with specific cellular messenger RNA transcripts
Source: PLoS Pathog. 2021 May 18;17(5):e1009589. doi: 10.1371/journal.ppat.1009589 (PMC8162694; doi:10.1371/journal.ppat.1009589)
Supplement: S2 Table — (DOCX) [file ppat.1009589.s007.docx]

| Primer name | Primer sequence (5´-3´) |
| --- | --- |
| InvRiL19 | /5Phos/rArGrArUrCrGrGrArArGrArGrCrArCrA rCrGrUrC/3SpC3/ |
| InvRand3Tr3 | 5Phos/NNNNNNNNNNAGATCGGAAGA GCGTCGTGT/3SpC3/ |
| InvAR17 | CAGACGTGTGCTCTTCCGA |
| D701: | CAAGCAGAAGACGGCATACGAGATCGAGTAATGTGACTGGAGTTCAGACGTGTGCTCTTCCGATC |
| D702: | CAAGCAGAAGACGGCATACGAGATTCTCCGGAGTGACTGGAGTTCAGACGTGTGCTCTTCCGATC |
| D703: | CAAGCAGAAGACGGCATACGAGATAATGAGCGGTGACTGGAGTTCAGACGTGTGCTCTTCCGATC |
| D704: | CAAGCAGAAGACGGCATACGAGATGGAATCTCGTGACTGGAGTTCAGACGTGTGCTCTTCCGATC |
| D705: | CAAGCAGAAGACGGCATACGAGATTTCTGAATGTGACTGGAGTTCAGACGTGTGCTCTTCCGATC |
| D706: | CAAGCAGAAGACGGCATACGAGATACGAATTCGTGACTGGAGTTCAGACGTGTGCTCTTCCGATC |
| D707: | CAAGCAGAAGACGGCATACGAGATAGCTTCAGGTGACTGGAGTTCAGACGTGTGCTCTTCCGATC |
| D708: | CAAGCAGAAGACGGCATACGAGATGCGCATTAGTGACTGGAGTTCAGACGTGTGCTCTTCCGATC |
| D709: | CAAGCAGAAGACGGCATACGAGATCATAGCCGGTGACTGGAGTTCAGACGTGTGCTCTTCCGATC |
| D710: | CAAGCAGAAGACGGCATACGAGATTTCGCGGAGTGACTGGAGTTCAGACGTGTGCTCTTCCGATC |
| D711: | CAAGCAGAAGACGGCATACGAGATGCGCGAGAGTGACTGGAGTTCAGACGTGTGCTCTTCCGATC |
| D712: | CAAGCAGAAGACGGCATACGAGATCTATCGCTGTGACTGGAGTTCAGACGTGTGCTCTTCCGATC |
| D501: | AATGATACGGCGACCACCGAGATCTACACTATAGCCTACACTCTTTCCCTACACGACGCTCTTCCGATCT |
| D502: | AATGATACGGCGACCACCGAGATCTACACATAGAGGCACACTCTTTCCCTACACGACGCTCTTCCGATCT |

**S2 Table: Primers used for seCLIP-seq**
