# Supplementary material for: Respiratory syncytial virus M2-1 protein associates non-specifically with viral messenger RNA and with specific cellular messenger RNA transcripts
Source: PLoS Pathog. 2021 May 18;17(5):e1009589. doi: 10.1371/journal.ppat.1009589 (PMC8162694; doi:10.1371/journal.ppat.1009589)
Supplement: S3 Table — (DOCX) [file ppat.1009589.s008.docx]

| Gene of interest | Forward primer sequence (5´-3´) | Reverse primer sequence (5´-3´) | Ta |
| --- | --- | --- | --- |
| RSV N | ATGGGAGAGGTAGCTCCAGA | AGCTCTCCTAATCACGGCTG | 60 |
| CANX | GATGGGGCCTGAAGAAAGCT | ATCCGGTTGAGGTGCATCAG | 60 |
| CD44 | CACACCCTCCCCTCATTCAC | TGTCCCTGTTGTCGAATGGG | 60 |
| CXCL5 | TGTGTTGAGAGAGCTGCGTT | CGTTCTTCAGGGAGGCTACC | 60 |
| IL6ST | ACACTTCGAGCACTGTCCAG | GTAGATCTTCTGGCCGCTCC | 60 |
| ITGA2 | ATTTCTTGAAGGCCCCGAGG | CGGATAGTGCCCTGATGACC | 60 |
| ITGAV | CAGGAGTTCCAAGAGCAGCA | TTTGCCATCAGAGCCACGAT | 60 |
| TGFBI | AACGGGAAGGCGATCATCTC | GTCAACCGCTCACTTCCAGA | 60 |
| DYNC1H1 | AGAAGTGAAGATGGGCGCAA | GATCCTCTCGCTGAAGTCGG | 60 |

**S3 Table: Primers used for RT-qPCR**
